# Insights into taurine therapy for periodontitis: Targeting osteocyte ferroptosis to mitigate obesity-exacerbated bone damage

**DOI:** 10.1016/j.redox.2026.104282

**Published:** 2026-06-30

**Authors:** Xin-Ge Chen, Xiao-Xue Zhu, Cui-Hua Cao, Yu-Zhe Chen, Yi-Ding Huo, Dian Gan, Dao-Kun Deng, Mei Xu, Hong-Lei Qu, Fa-Ming Chen, Bei-Min Tian, Xuan Li

**Affiliations:** a State Key Laboratory of Oral & Maxillofacial Reconstruction and Regeneration, National Clinical Research Center for Oral Diseases, Shaanxi International Joint Research Center for Oral Diseases, Department of Periodontology, School of Stomatology, The Fourth Military Medical University, Xi'an, Shaanxi, 710032, China; b Military Medical Innovation Center, The Fourth Military Medical University, Xi'an, Shaanxi, China

**Keywords:** Periodontitis, Obesity, Osteocyte ferroptosis, Taurine, Alveolar bone loss

## Abstract

Obesity is a major systemic risk factor for periodontitis and is associated with increased alveolar bone destruction. However, the mechanisms by which obesity-related metabolic stress aggravates periodontal bone loss remain poorly understood. This study sought to elucidate whether osteocyte ferroptosis represents a key mechanistic driver of obesity-exacerbated periodontal bone destruction and whether taurine alleviates this pathology, at least in part, by suppressing ferroptosis. We established a mouse model of diet-induced obesity with experimental periodontitis and an *in vitro* MLO-Y4 osteocyte model exposed to palmitic acid and lipopolysaccharide to recapitulate the combined lipotoxic and inflammatory microenvironment of obesity-associated periodontitis. Compared with periodontitis alone, obesity significantly exacerbated alveolar bone loss, osteocyte-mediated remodeling imbalance, and systemic inflammatory burden. RNA-sequencing and molecular analyses revealed increased osteocyte ferroptosis, characterized by severe lipid peroxidation, iron dyshomeostasis, and impaired antioxidant defense. Notably, obese mice with periodontitis exhibited systemic taurine deficiency. Taurine supplementation markedly attenuated alveolar bone loss, restored osteocyte-mediated remodeling balance, and robustly suppressed osteocyte ferroptosis both *in vivo* and *in vitro*. Importantly, co-administration of the ferroptosis activator erastin partially abolished the protective effects of taurine, further supporting a ferroptosis-dependent protective mechanism. Our findings reveal osteocyte ferroptosis as a previously unrecognized pathogenic mechanism in obesity-exacerbated periodontal bone destruction. Additionally, we identify taurine as a promising therapeutic strategy targeting osteocyte ferroptosis, offering new mechanistic insights and translational potential for managing obesity-associated periodontitis.

## Highlights

• Obesity worsens periodontitis-associated alveolar bone loss and osteocyte dysfunction.
• Osteocyte ferroptosis drives obesity-exacerbated periodontal bone loss.
• Taurine suppresses osteocyte ferroptosis and restores bone remodeling signals.

## Graphical abstract

**Schematic illustration of the proposed mechanism by which taurine mitigates obesity-exacerbated alveolar bone destruction in periodontitis.** In ligature- and *Porphyromonas gingivalis* (*P. gingivalis*)-induced periodontitis, obesity further enhances osteocyte ferroptosis in alveolar bone, characterized by increased Fe^2+^ accumulation, elevated lipid ROS production, and mitochondrial shrinkage. Enhanced osteocyte ferroptosis serves as a key mechanism underlying obesity-exacerbated periodontal bone loss and is accompanied by increased systemic inflammation and pro-osteoclastogenic activity. Taurine alleviates obesity-exacerbated alveolar bone destruction, at least in part, by suppressing osteocyte ferroptosis; this protective effect is accompanied by reduced systemic inflammation and pro-osteoclastogenic activity.

**Figure:**
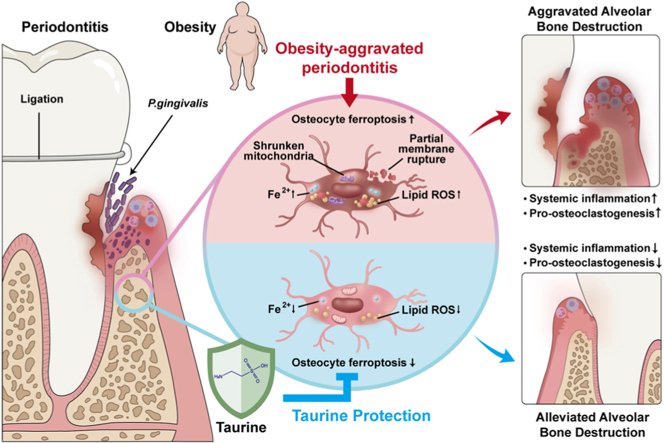

## 1 Introduction

Periodontitis is a chronic inflammatory disease of tooth-supporting tissues and is a major cause of tooth loss in adults [1]. Its irreversible progression is driven by alveolar bone (AB) resorption, the central pathological event underlying permanent periodontal destruction [[2], [3], [4]]. In addition to its local pathological manifestations, periodontitis is increasingly recognized as a disease closely associated with systemic health conditions [5]. Among these factors, obesity has emerged as a significant and increasingly prevalent aggravating factor [6,7]. Clinical and experimental studies have indicated that obesity increases susceptibility to periodontitis and worsens the degradation of periodontal tissues, resulting in greater attachment loss, more severe alveolar bone destruction, and a poorer treatment response [[8], [9], [10], [11]]. Although these effects have been attributed to chronic low-grade inflammation and metabolic dysregulation [12,13], the precise cellular and molecular mechanisms within alveolar bone through which obesity-driven metabolic disturbances are translated into accelerated bone destruction remain poorly defined.

Ferroptosis is an iron-dependent form of regulated cell death characterized by excessive lipid peroxidation and impaired antioxidant defenses [14,15]. It has recently been implicated in metabolic disorders, inflammatory tissue injury, and skeletal pathology [[16], [17], [18]]. Obesity induces lipid overload, oxidative imbalance, and altered iron homeostasis [19,20], compounded by the persistent local inflammatory stress of periodontitis, and may create a microenvironment that favors ferroptosis in alveolar bone. Among bone-resident cells, osteocytes are of particular interest because they are central regulators of bone remodeling, and their injury or death is closely linked to periodontal bone loss [21,22]. Osteocyte ferroptosis has recently emerged as a contributor to periodontal injury and inflammatory bone loss [23,24], suggesting that it may serve as a key mechanism by which obesity-associated metabolic stress amplifies alveolar bone destruction. However, whether this pathway directly contributes to obesity-exacerbated alveolar bone loss during periodontitis has not been explored.

After identifying a potential pathogenic mechanism, the next step was to explore a candidate molecule capable of modulating the overlapping metabolic, oxidative, and inflammatory dysregulation that characterizes obesity-exacerbated periodontitis. Taurine is of particular interest in this context, as previous studies have demonstrated its protective effects in both metabolic disorders and periodontitis, including anti-inflammatory, antioxidant, and tissue-protective actions [[25], [26], [27], [28]]. Recent evidence further suggests that taurine may possess anti-ferroptotic potential in several disease models through regulation of redox homeostasis, antioxidant defense, and iron metabolism [[29], [30], [31]]. However, whether taurine can attenuate obesity-exacerbated alveolar bone loss in periodontitis, particularly through suppression of osteocyte ferroptosis, remains unknown.

In this study, we established an obese mouse model with periodontitis by combining diet-induced obesity with silk ligation and topical application of a *Porphyromonas gingivalis* (*P. gingivalis*) suspension [[32], [33], [34]]. In parallel, MLO-Y4 osteocyte-like cells were treated with palmitic acid (PA) to mimic obesity-related lipotoxic stress and with *P. gingivalis*-derived lipopolysaccharide (LPS) to mimic the periodontitis-related inflammatory microenvironment [35,36]. Using these combined *in vivo* and *in vitro* approaches, we aimed to determine whether osteocyte ferroptosis contributes to obesity-exacerbated periodontal bone destruction. We further investigated whether taurine could serve as a therapeutic agent for obesity-exacerbated periodontitis and whether its protective effects against alveolar bone loss were mediated by suppressing osteocyte ferroptosis.

By addressing these questions, this study may provide new insight into the pathogenic link between obesity-related metabolic stress and periodontal bone destruction, while also supporting ferroptosis modulation, particularly through the use of taurine as a potential therapeutic strategy for obesity-associated periodontitis.

## 2 Results

### 2.1 Obesity aggravates alveolar bone damage, osteocyte dysfunction, and systemic inflammation in mice with periodontitis

**Fig. 1:**
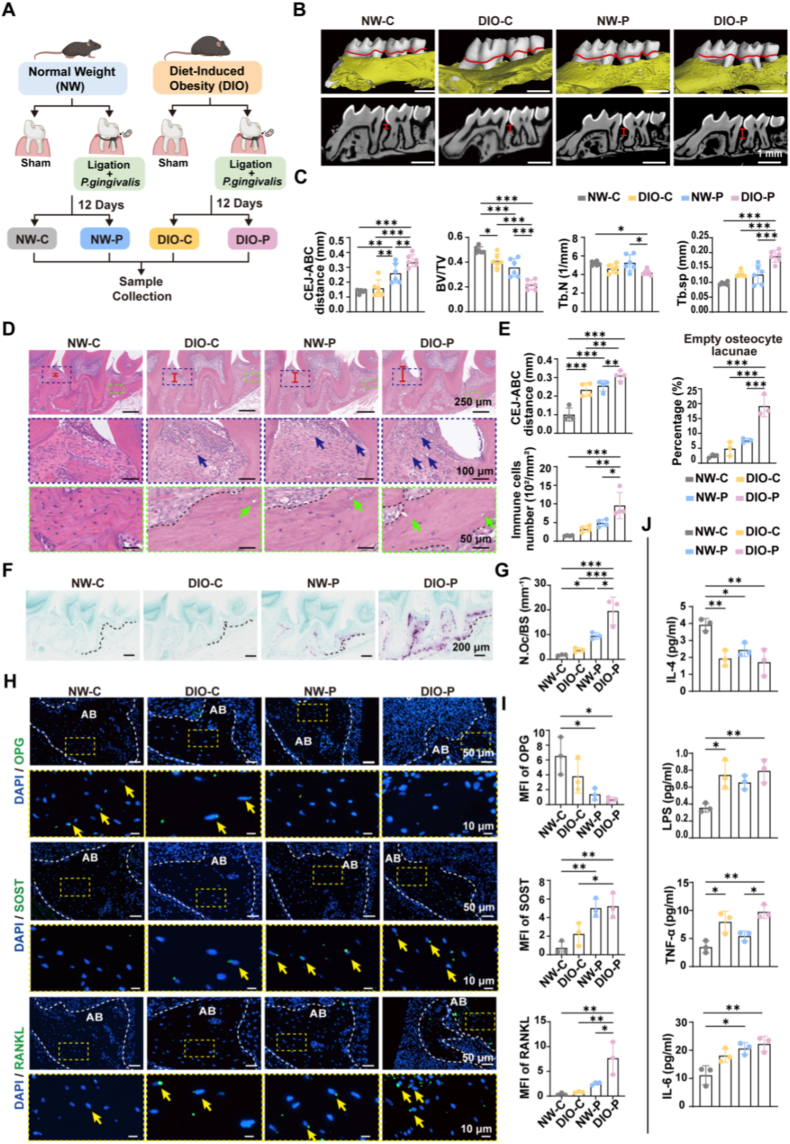
Effects of obesity on alveolar bone damage, osteocyte function, and systemic inflammation in a mouse model of experimental periodontitis. **A** Schematic of the experimental workflow. Mice were assigned to normal-weight (NW) or diet-induced obese (DIO) groups and further divided into control (C) or experimental periodontitis (P) subgroups, yielding 4 groups: NW-C (normal weight control), NW-P (normal weight with periodontitis), DIO-C (diet-induced obesity control), and DIO-P (diet-induced obesity with periodontitis). **B** Representative micro-CT images of maxillary alveolar bone are shown as 3D reconstructions (top panels) and sagittal sections (bottom panels). Red lines mark the cementoenamel junction (CEJ), and red vertical segments indicate the CEJ–alveolar bone crest (ABC) distance. Scale bar, 1 mm. **C** Quantitative assessment of the CEJ–ABC distance, bone volume/tissue volume (BV/TV), trabecular number (Tb.N) and trabecular separation (Tb.Sp) in the interdental region between the first and second molars. **D** Representative images of hematoxylin and eosin (H&E)-stained sagittal sections of periodontal tissues. Red vertical line segments indicate the CEJ–ABC distance. The blue dashed boxes denote enlarged images of the mesial gingiva adjacent to the second molar, and the green dashed boxes denote enlarged images of the distal alveolar bone adjacent to the second molar. The blue arrows indicate infiltrating immune cells, and the green arrows indicate empty osteocyte lacunae. Black dashed lines outline the bone boundaries. Scale bars: 250 μm (top panels), 100 μm (middle panels), and 50 μm (bottom panels). **E** Histomorphometric quantification of the CEJ–ABC distance, the number of infiltrating immune cells per unit area, and the percentage of empty osteocyte lacunae. **F** Representative sagittal sections of the second molar region after TRAP staining. The black dashed lines outline the alveolar bone surface used for the osteoclast assessment. Scale bar, 200 μm. **G** Quantification of TRAP-positive osteoclasts reported as the number of osteoclasts per bone surface (N.Oc/BS) at the distal alveolar bone of the second molar. **H** Representative images of immunofluorescence (IF) staining for osteoprotegerin (OPG), sclerostin (SOST), and receptor activator of nuclear factor-κB ligand (RANKL) in the alveolar bone (AB). Target proteins are presented in green, and nuclei are counterstained with DAPI (blue). The yellow dashed boxes indicate enlarged bone regions. Yellow arrows indicate positively stained areas. The white dashed lines demarcate the bone boundaries. Scale bars, 50 μm and 10 μm. **I** Quantitative analysis of OPG, SOST, and RANKL expression, and the results are reported as the mean fluorescence intensity (MFI). **J** Serum levels of anti-inflammatory cytokines (IL-4), endotoxin (LPS), and proinflammatory cytokines (TNF-α and IL-6) were measured using a Luminex multiplex assay. The data are shown as the means ± SDs. Differences among groups were analyzed using one-way analysis of variance (ANOVA) followed by Tukey's multiple comparisons test. ∗*P* < 0.05, ∗∗*P* < 0.01, and ∗∗∗*P* < 0.001. *n* = 3–6 mice per group.

We established a diet-induced obesity (DIO) model by feeding mice a high-fat diet (HFD) for 12 weeks. Compared with mice fed a normal diet (ND), HFD-fed mice showed progressive weight gain (Supplementary Fig. S1A); elevated blood glucose levels at weeks 8 and 12 (Supplementary Fig. S1B); impaired glucose tolerance at week 12, as reflected by higher glucose levels during the oral glucose tolerance test (OGTT), and an increased area under the curve (AUC) (Supplementary Fig. S1C); and significantly increased fat mass and body fat percentage (Supplementary Fig. S1D). These data confirmed the successful establishment of the DIO model. Normal-weight (NW) and DIO mice were then assigned to control (NW-C and DIO-C) or periodontitis groups (NW-P and DIO-P). Experimental periodontitis was induced by silk ligation combined with the topical application of *P. gingivalis* (Fig. 1A).

Micro-CT showed alveolar bone loss in both periodontitis groups (NW-P and DIO-P), which was more severe in DIO-P mice than in NW-P mice (Fig. 1B). Accordingly, DIO-P mice presented a greater cementoenamel junction (CEJ) to alveolar bone crest (ABC) distance, lower bone volume/tissue volume (BV/TV) and trabecular number (Tb.N), and greater trabecular separation (Tb.Sp) than NW-P mice (Fig. 1C), indicating that obesity aggravated periodontitis-associated deterioration of the alveolar bone microarchitecture. The histological analysis confirmed these findings (Fig. 1D–G). Hematoxylin and eosin (H&E) staining showed more extensive periodontal tissue destruction in DIO-P mice than in NW-P mice, and was characterized by a greater CEJ–ABC distance, increased inflammatory cell infiltration, and a higher proportion of empty osteocyte lacunae, suggesting more severe osteocyte damage in obese mice (Fig. 1D and E). Tartrate-resistant acid phosphatase (TRAP) staining and the histomorphometric analysis further demonstrated greater osteoclast accumulation along the alveolar bone surface (BS) in DIO-P mice, together with a significantly greater osteoclast number per bone surface (N.Oc/BS) than in NW-P mice (Fig. 1F and G).

To better characterize osteocyte dysfunction in obesity-exacerbated periodontitis, we used immunofluorescence (IF) staining to assess the alveolar bone expression of remodeling-related proteins, including osteoprotegerin (OPG), sclerostin (SOST), and receptor activator of nuclear factor-κB ligand (RANKL). Both periodontitis groups (NW-P and DIO-P) showed reduced OPG expression, increased SOST and RANKL expression compared with controls (Fig. 1H and I). Notably, compared with that in NW-P mice, only RANKL expression was further increased in DIO-P mice (Fig. 1H and I). These findings indicate that obesity selectively enhances local pro-osteoclastogenic signaling in an experimental periodontitis model.

A multiplex analysis of serum samples was subsequently performed to evaluate the effect of obesity on systemic inflammation under periodontitis conditions. The periodontitis groups (NW-P and DIO-P) showed increased interleukin-6 (IL-6) and decreased interleukin-4 (IL-4) levels, whereas the DIO groups (DIO-C and DIO-P) presented higher circulating LPS levels than NW-C mice (Fig. 1J). Specifically, among the periodontitis groups (NW-P and DIO-P), tumor necrosis factor-α (TNF-α) levels were significantly higher in DIO-P mice than in NW-P mice (Fig. 1J), indicating that obesity further amplifies systemic inflammation in mice with periodontitis. Together, these findings indicate that obesity exacerbates periodontitis-associated alveolar bone destruction, osteocyte dysfunction, and systemic inflammation.

### 2.2 Obesity aggravates osteocyte ferroptosis in the alveolar bone surrounding periodontitis-affected molars in mice

**Fig. 2:**
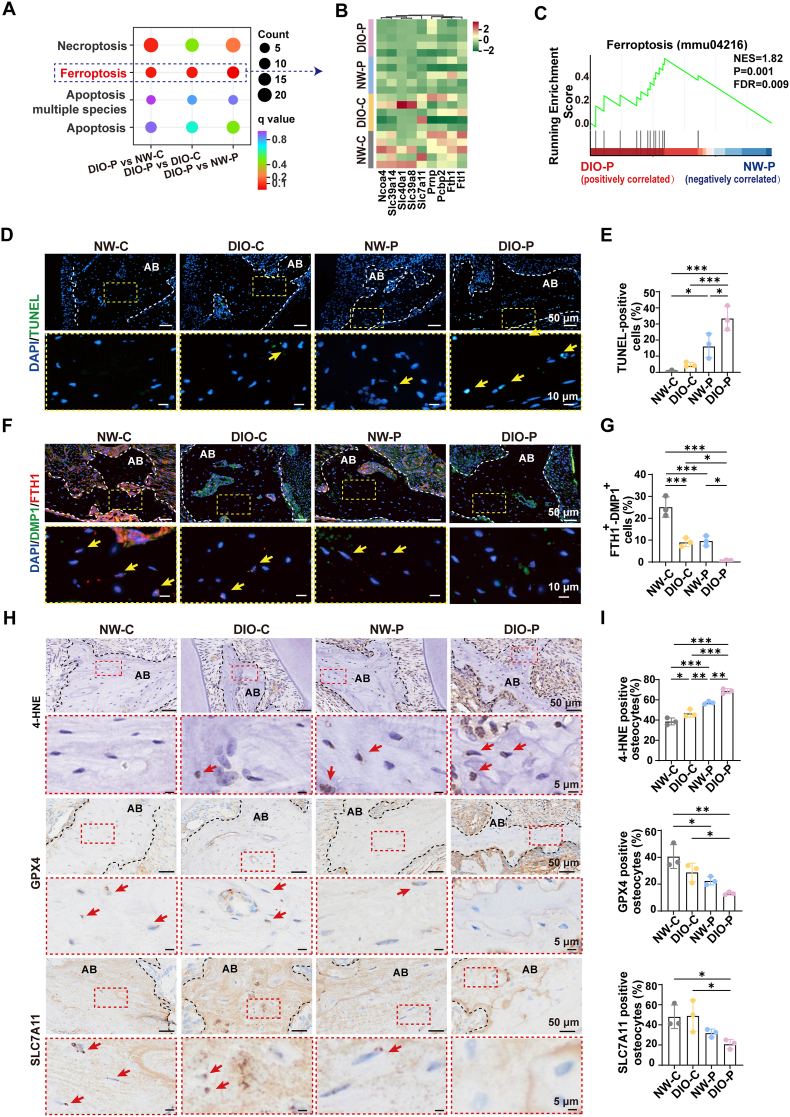
**Effects of obesity on osteocyte ferroptosis in alveolar bone surrounding periodontitis-affected molars in mice. A** Bubble plot of the results of the Kyoto Encyclopedia of Genes and Genomes (KEGG) pathway enrichment analysis for upregulated differentially expressed genes (DEGs) in DIO-P mice compared with the other three groups (NW-C, DIO-C, and NW-P). The ferroptosis pathway was significantly enriched (highlighted in red). The dot color indicates the *q* value (adjusted *P* value), and the dot size reflects the number of enriched genes. **B** Heatmap showing the expression profiles of ferroptosis pathway-related DEGs across the groups (NW-C, DIO-C, NW-P, and DIO-P). The data are presented as row-scaled Z scores of fragments per kilobase of transcript per million mapped reads (FPKM) values. **C** Gene set enrichment analysis (GSEA) of the ferroptosis gene set (mmu04216) comparing the DIO-P and NW-P groups. NES, normalized enrichment score; *P*, *P* value; FDR, false discovery rate. **D** Representative fluorescence images of TUNEL-positive osteocytes (green) in AB. Nuclei were counterstained with DAPI (blue). Yellow arrows denote TUNEL-positive cells, and white dashed lines demarcate the bone boundaries. Scale bars, 50 μm and 10 μm. **E** Quantitative assessment of the percentage of TUNEL-positive osteocytes in AB. **F** Representative images of IF staining showing the colocalization of ferritin heavy chain 1 (FTH1; red) with the osteocyte marker dentin matrix protein-1 (DMP1; green) in AB. Yellow arrows indicate FTH1^+^DMP1^+^ cells, and white dashed lines demarcate the bone boundaries. Scale bars, 50 μm and 10 μm. **G** Quantitative assessment of the percentage of FTH1^+^DMP1^+^ cells in AB. **H** Representative immunohistochemical (IHC) images of staining for 4-hydroxynonenal (4-HNE), glutathione peroxidase 4 (GPX4), and the cystine/glutamate transporter (xCT/SLC7A11) in AB. Red arrows indicate positively stained osteocytes within the bone matrix, and black dashed lines demarcate the bone boundaries. Scale bars, 50 μm and 5 μm. **I** Quantification of the percentages of 4-HNE-, GPX4-, and SLC7A11-positive osteocytes in AB. The data are shown as the means ± SDs. Differences among groups were analyzed using one-way ANOVA followed by Tukey's multiple-comparisons test. ∗*P* < 0.05, ∗∗*P* < 0.01, ∗∗∗*P* < 0.001. *n* = 3–5 mice per group.

We performed RNA sequencing (RNA-seq) on soft tissue-denuded alveolar bone surrounding the ligated molars from NW-C, DIO-C, NW-P, and DIO-P mice to explore the molecular pathways underlying the obesity-exacerbated phenotype. Volcano plots revealed a substantial number of differentially expressed genes (DEGs) in the DIO-P group compared with the NW-C, DIO-C, and NW-P groups (Supplementary Fig. S2). The Kyoto Encyclopedia of Genes and Genomes (KEGG) enrichment analysis of upregulated DEGs revealed that, among the cell death-related pathways identified, ferroptosis was the only pathway consistently and significantly enriched across all three pairwise comparisons between DIO-P and the other groups (NW-C, DIO-C, and NW-P) (Fig. 2A). In line with this finding, the heatmap further showed a distinct expression pattern of ferroptosis-related genes in DIO-P mice (Fig. 2B). Gene set enrichment analysis (GSEA) further showed the significant positive enrichment of the ferroptosis gene set in DIO-P mice compared with NW-P mice (Fig. 2C; NES = 1.82, *P* = 0.001, FDR = 0.009). qRT-PCR validation revealed that *Gpx4* expression was lower in DIO-P mice than in NW-C, DIO-C, and NW-P mice (Supplementary Fig. S3), corroborating the RNA-seq results. These transcriptomic findings indicate that obesity specifically enhances ferroptosis-related signaling in periodontitis-affected alveolar bone.

We assessed osteocyte damage and the expression of ferroptosis-related markers in the alveolar bone surrounding periodontitis-affected molars to determine whether obesity aggravates osteocyte ferroptosis in this region. TUNEL staining showed increased numbers of TUNEL-positive osteocytes in both periodontitis groups (NW-P and DIO-P), with the strongest signal observed in DIO-P mice. Quantification revealed that DIO-P mice had a significantly greater proportion of TUNEL-positive osteocytes than NW-P mice (Fig. 2D and E), indicating aggravated osteocyte death under obese conditions. As *ferritin heavy chain 1 gene* (*fth1*) emerged as a ferroptosis-related DEG according to the results of the RNA-seq analysis (Fig. 2B), we next examined Ferritin heavy chain 1 (FTH1) expression in osteocytes by performing co-IF staining for FTH1 and DMP1 (osteocyte marker). Compared with NW-P mice, DIO-P mice had a lower proportion of FTH1^+^DMP1^+^ cells (Fig. 2F and G), suggesting that the iron-storage capacity of osteocytes is impaired in the context of obesity-associated periodontitis. We further evaluated the expression of additional ferroptosis-related markers, including 4-hydroxynonenal (4-HNE), glutathione peroxidase 4 (GPX4), and solute carrier family 7 member 11 (xCT/SLC7A11), by performing immunohistochemistry (IHC). The percentage of 4-HNE-positive osteocytes was increased in DIO-P mice compared with NW-P mice (Fig. 2H and I). Meanwhile, GPX4 and SLC7A11 expression tended to decrease in DIO-P mice (Fig. 2H and I). Collectively, these findings indicate that obesity aggravates osteocyte ferroptosis in the alveolar bone of mice with periodontitis.

### 2.3 Inhibition of osteocyte ferroptosis attenuates alveolar bone damage, osteocyte dysfunction, and systemic inflammation in obese mice with periodontitis

**Fig. 3:**
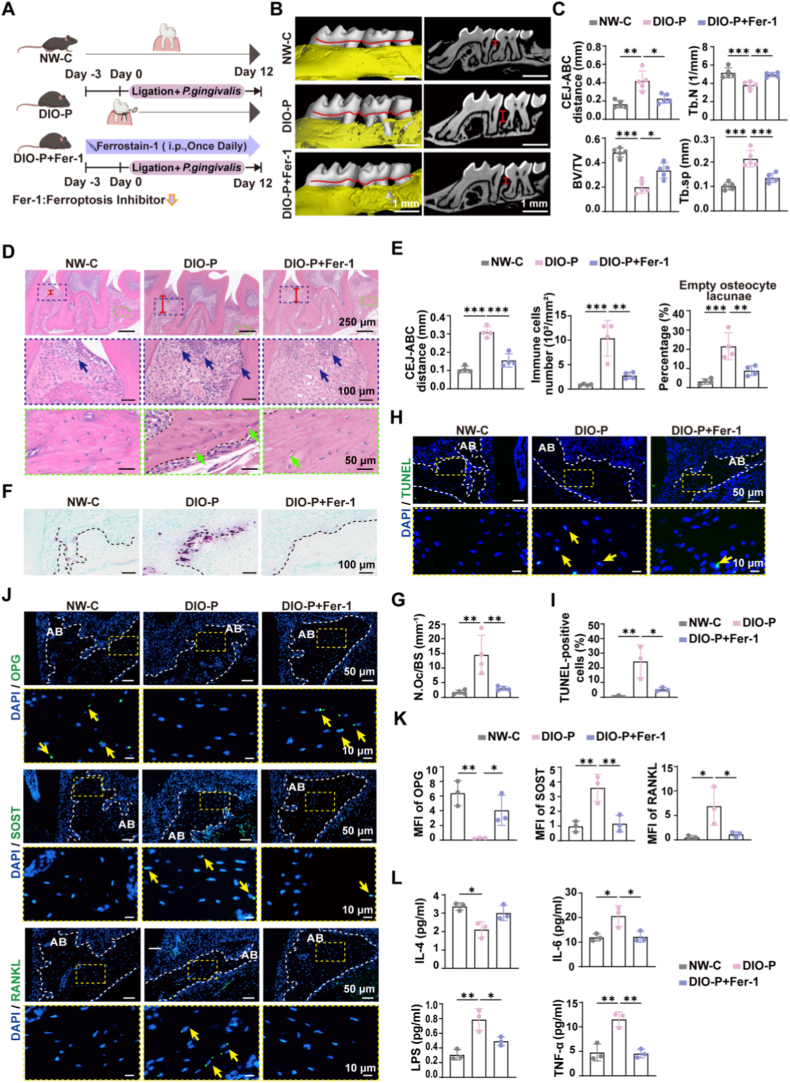
Roles of osteocyte ferroptosis in alveolar bone damage, osteocyte dysfunction, and the levels of systemic inflammatory markers in obese mice with periodontitis. A Schematic illustration of the experimental workflow. Mice were assigned to three groups: NW-C (normal weight control), DIO-P (diet-induced obese mice with periodontitis) and DIO-P mice treated with ferrostatin-1 (DIO-P + Fer-1; 10 mg/kg, i.p., once daily). **B** Representative micro-CT scans of maxillary alveolar bone shown as 3D reconstructions and sagittal views on day 12. Red lines mark the CEJ, and red line segments indicate the CEJ–ABC distance. Scale bar, 1 mm. **C** Quantitative micro-CT analysis of the CEJ–ABC distance, BV/TV, Tb.N and Tb.Sp in the interdental region between the first and second molars. **D** Representative H&E-stained images. Red vertical line segments indicate the CEJ–ABC distance. The blue dashed boxes denote enlarged images of the mesial gingiva adjacent to the second molar, and the green dashed boxes denote enlarged images of the distal alveolar bone adjacent to the second molar. The blue arrows indicate infiltrating immune cells, and the green arrows indicate empty osteocyte lacunae. Black dashed lines outline the bone boundaries. Scale bars, 250 μm (top panels), 100 μm (middle panels), and 50 μm (bottom panels). **E** Histomorphometric quantification of the CEJ–ABC distance, the number of infiltrating immune cells per unit area, and the percentage of empty osteocyte lacunae. **F** Representative images of TRAP-stained distal alveolar bone around the second molar. The black dashed lines outline the BS used for the osteoclast assessment. Scale bar, 100 μm. **G** Quantification of TRAP-positive osteoclasts expressed as N.Oc/BS in the distal alveolar bone of the second molar. **H** Representative fluorescence images of TUNEL-positive osteocytes (green) in AB. Nuclei were counterstained with DAPI (blue). Yellow dashed boxes indicate enlarged bone regions, and white dashed lines demarcate bone boundaries. Yellow arrows denote TUNEL-positive cells. Scale bars, 50 μm and 10 μm. **I** Quantitative assessment of the percentage of TUNEL-positive osteocytes in AB. **J** Representative images of IF staining showing OPG, SOST, and RANKL expression in AB. Target proteins are presented in green, and nuclei are counterstained with DAPI (blue). Yellow dashed boxes mark enlarged bone regions, and white dashed lines demarcate bone boundaries. Yellow arrows indicate positively stained areas. Scale bars, 50 μm and 10 μm. **K** Quantification analysis of the MFIs of OPG, SOST, and RANKL. **L** Circulating IL-4, LPS, TNF-α, and IL-6 levels were detected using a Luminex multiplex assay. The data are shown as the means ± SDs. Differences among groups were analyzed using one-way ANOVA followed by Dunnett's multiple-comparisons test. ∗*P* < 0.05, ∗∗*P* < 0.01, and ∗∗∗*P* < 0.001. *n* = 3–5 mice per group.

DIO mice with experimental periodontitis were treated with ferrostatin-1 (DIO-P + Fer-1) to determine whether pharmacological inhibition of ferroptosis could mitigate obesity-exacerbated alveolar bone damage. Untreated DIO-P and NW-C mice served as control groups (Fig. 3A). IF and IHC staining were performed to detect ferroptosis-related markers and evaluate whether Fer-1 treatment successfully inhibited osteocyte ferroptosis in AB. Compared with untreated DIO-P mice, Fer-1-treated mice exhibited restored FTH1 expression, decreased 4-HNE accumulation, and increased GPX4 and SLC7A11 levels (Supplementary Fig. S4), indicating the effective suppression of osteocyte ferroptosis in AB. Micro-CT assessment demonstrated pronounced alveolar bone resorption and impaired trabecular architecture in DIO-P mice, whereas Fer-1 administration substantially alleviated these abnormalities. Quantitative measurements further showed that DIO-P + Fer-1 mice had a significantly reduced CEJ–ABC distance, increased BV/TV and Tb.N, and decreased Tb.Sp compared with untreated DIO-P mice (Fig. 3B and C). The histological findings were consistent with the micro-CT results, further indicating that ferroptosis inhibition alleviated obesity-associated alveolar bone destruction in a periodontitis model (Fig. 3D–G). H&E staining showed a shorter CEJ–ABC distance, less inflammatory cell infiltration, and fewer empty osteocyte lacunae in DIO-P + Fer-1 mice than in untreated DIO-P mice, suggesting attenuated osteocyte damage after Fer-1 treatment (Fig. 3D and E). Consistent with these findings, TUNEL staining revealed a marked reduction in the proportion of TUNEL-positive osteocytes in DIO-P + Fer-1 mice (Fig. 3H and I), corroborating the reduction in osteocyte death upon ferroptosis inhibition. TRAP staining and the histomorphometric analysis further demonstrated reduced osteoclast accumulation along the alveolar bone surface and a significantly lower N.Oc/BS after Fer-1 treatment (Fig. 3F and G).

We investigated whether ferroptosis inhibition influences osteocyte function by examining the expression of key bone remodeling regulators (OPG, SOST, and RANKL). Compared with untreated DIO-P mice, Fer-1 treatment decreased RANKL and SOST expression and increased OPG levels in DIO-P mice (Fig. 3J and K), indicating a partial restoration of osteocyte-mediated bone remodeling balance after ferroptosis inhibition. Serum was subsequently analyzed to explore whether ferroptosis inhibition modulated systemic inflammation in obese mice with periodontitis. Compared with untreated DIO-P mice, Fer-1 treatment significantly reduced the circulating levels of LPS, TNF-α, and IL-6 (Fig. 3L), indicating that ferroptosis inhibition primarily attenuated systemic proinflammatory alterations in an obesity-associated periodontitis model. Overall, these findings indicate that the pharmacological inhibition of osteocyte ferroptosis protects against obesity-exacerbated alveolar bone damage, ameliorates osteocyte-associated remodeling dysfunction, and alleviates systemic inflammatory burden in mice with periodontitis.

### 2.4 Ferroptosis mediates cellular dysfunction and cytokine production in MLO-Y4 osteocytes upon PA and LPS exposure

**Fig. 4:**
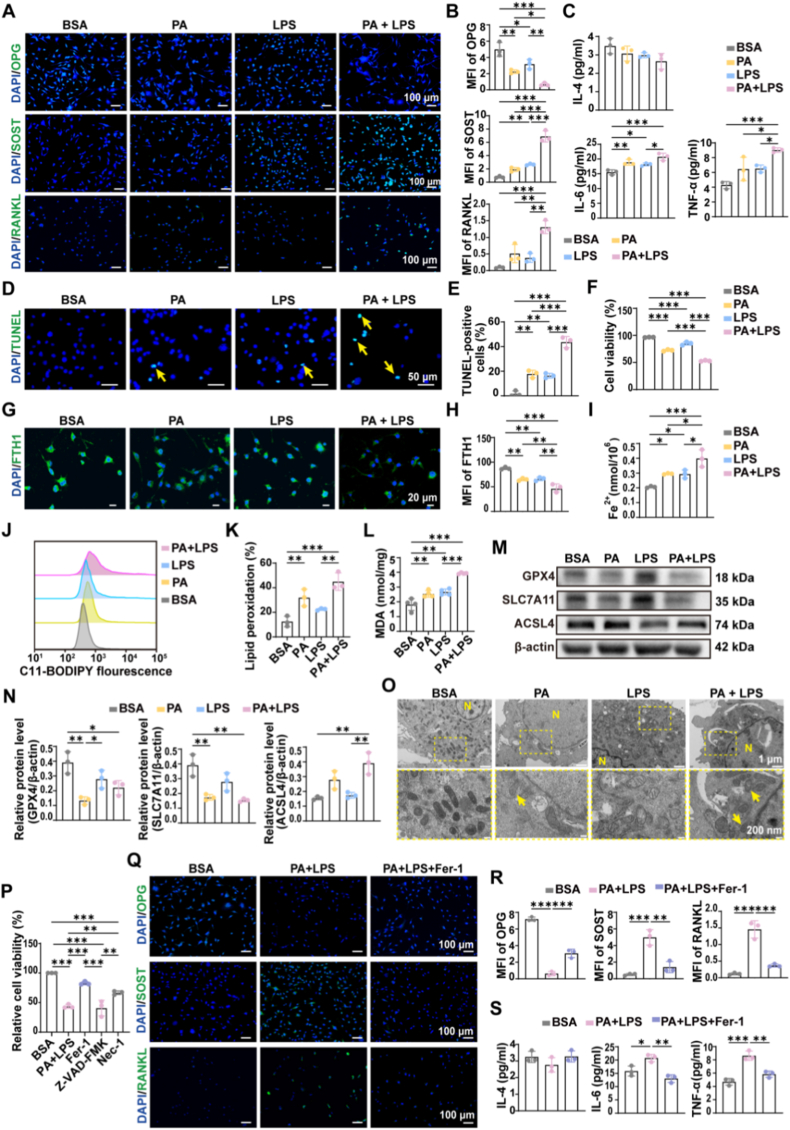
**Effects of PA and/or LPS stimulation on osteocyte function, cytokine production, and ferroptosis in MLO-Y4 osteocytes. A** Representative images of IF staining for OPG, SOST, and RANKL in MLO-Y4 cells. The target proteins are presented in green, and nuclei are counterstained with DAPI (blue). Scale bar, 100 μm. **B** Quantification analysis of the MFIs of OPG, SOST, and RANKL. **C** IL-4, IL-6, and TNF-α levels in culture supernatants detected by a Luminex multiplex assay. **D** Representative fluorescence images of TUNEL (green) staining. Nuclei were counterstained with DAPI (blue). Yellow arrows indicate TUNEL-positive cells. Scale bar, 50 μm. **E** Quantitative assessment of TUNEL-positive cells. **F** Cell viability of MLO-Y4 osteocytes was determined by CCK-8 assay. **G** Representative images of IF staining for FTH1 (green) in MLO-Y4 cells. Nuclei were counterstained with DAPI (blue). Scale bar, 20 μm. **H** MFI analysis of FTH1. **I** Intracellular Fe^2+^ content determined using a colorimetric assay and normalized to cell number (nmol/10^6^ cells). **J** Representative flow cytometry plots of lipid ROS generation detected by C11-BODIPY staining. **K** Quantification of lipid peroxidation, expressed as the percentage of C11-BODIPY-positive cells. **L** Malondialdehyde (MDA) levels were measured by performing a colorimetric assay and normalized to the total protein content (nmol/mg). **M** Representative western blots showing GPX4, SLC7A11, and ACSL4 expression. **N** Densitometric quantification of GPX4, SLC7A11, and ACSL4 protein expression after normalization to β-actin. **O** Representative transmission electron microscopy (TEM) images of the perinuclear region showing mitochondrial and nuclear ultrastructural alterations. Yellow dashed boxes indicate enlarged views highlighting the mitochondrial ultrastructure, and yellow arrows indicate characteristic ferroptosis-like changes. Scale bars, 1 μm and 200 nm. **P** Viability of MLO-Y4 cells treated with PA + LPS in the presence or absence of ferrostatin-1 (Fer-1, 10 μM), Z-Val-Ala-Asp fluoromethylketone (Z-VAD-FMK; 20 μM), or necrostatin-1 (Nec-1, 20 μM). **Q** Representative IF images showing OPG, SOST, and RANKL expression in MLO-Y4 cells after Fer-1 treatment. The target proteins are presented in green, and nuclei are counterstained with DAPI (blue). Scale bar, 100 μm. **R** Quantification analysis of the MFIs of OPG, SOST, and RANKL. **S** IL-4, IL-6, and TNF-α levels in culture supernatants from cells exposed to PA + LPS with or without Fer-1. The data are shown as the means ± SDs. Differences among groups were analyzed using one-way ANOVA followed by Tukey's or Dunnett's multiple comparisons test. ∗*P* < 0.05, ∗∗*P* < 0.01, and ∗∗∗*P* < 0.001. *n* = 3–4 independent experiments.

As a method to further validate these findings *in vitro*, MLO-Y4 osteocytes were challenged with PA and LPS to mimic obesity-associated lipotoxicity and periodontitis-related inflammatory stress, respectively. Whether lipotoxic stress can aggravate LPS-induced osteocyte dysregulation was first explored. Compared with LPS alone, PA + LPS treatment further decreased OPG expression while increasing SOST and RANKL immunofluorescence signals, indicating a more pronounced shift toward a proresorptive osteocytic phenotype (Fig. 4A and B). Compared with LPS alone, PA + LPS treatment further increased IL-6 and TNF-α secretion (Fig. 4C). These data suggest that PA primarily amplifies the LPS-induced proinflammatory output. Since PA markedly intensified LPS-induced osteocyte dysregulation, we next examined whether this effect was associated with ferroptosis. Compared with treatment with LPS alone, treatment with PA + LPS markedly increased the proportion of TUNEL-positive cells (Fig. 4D and E) accompanied by a further decline in cell viability determined by Cell Counting Kit-8 (CCK-8) analysis (Fig. 4F). In parallel, PA + LPS treatment aggravated ferroptosis-associated disturbances in iron homeostasis, as evidenced by reduced FTH1 expression together with increased intracellular labile Fe^2+^ levels (Fig. 4G–I). Moreover, compared with treatment with LPS alone, treatment with PA + LPS increased lipid peroxidation, evidenced by enhanced C11-BODIPY lipid ROS signals and MDA levels (Fig. 4J–L). Consistent with these findings, the ferroptosis-related marker acyl-CoA synthetase long-chain family member 4 (ACSL4) was significantly increased in cells receiving combined PA + LPS treatment relative to those treated with LPS alone (Fig. 4M and N). TEM provided further ultrastructural support for ferroptosis. Compared with treatment with LPS alone, PA + LPS treatment induced more pronounced mitochondrial shrinkage, higher membrane electron density, and loss or disrupted cristae, all of which are recognized morphological hallmarks of ferroptosis [37] (Fig. 4O). In contrast, obvious dilation of the perinuclear space, a feature more commonly associated with necroptosis was not observed [38]. Thus, the ultrastructural evidence favors ferroptosis over necroptosis, in PA + LPS-treated cells.

We compared the effects of pharmacological inhibitors of ferroptosis, apoptosis, and necroptosis on cell viability to further identify the cell death pathway involved in PA + LPS-induced osteocyte death. Upon PA + LPS stimulation, the inhibition of ferroptosis with Fer-1 restored cell viability more effectively than inhibition of apoptosis with Z-Val-Ala-Asp fluoromethylketone (Z-VAD-FMK) or the inhibition of necroptosis with necrostatin-1 (Nec-1) (Fig. 4P), suggesting that ferroptosis is a major contributor to osteocyte death under combined lipotoxic and inflammatory stress. Next, we assessed whether the pharmacological inhibition of ferroptosis could mitigate PA + LPS-induced osteocyte dysfunction. IF staining showed that, compared with PA + LPS treatment alone, Fer-1 cotreatment partially restored OPG expression and reduced SOST and RANKL signals (Fig. 4Q and R), indicating that ferroptosis inhibition mitigated the proresorptive shift in osteocyte-derived remodeling signals under combined lipotoxic and inflammatory stimulation. In addition, cytokine profiling showed that Fer-1 cotreatment attenuated PA + LPS-induced increases in IL-6 and TNF-α levels (Fig. 4S). Overall, these data support a role for ferroptosis in mediating osteocyte dysfunction and inflammatory cytokine release under combined PA and LPS stimulation *in vitro.*

### 2.5 Taurine alleviates obesity-exacerbated alveolar bone damage, osteocyte dysfunction, and systemic inflammation in mice with periodontitis

**Fig. 5:**
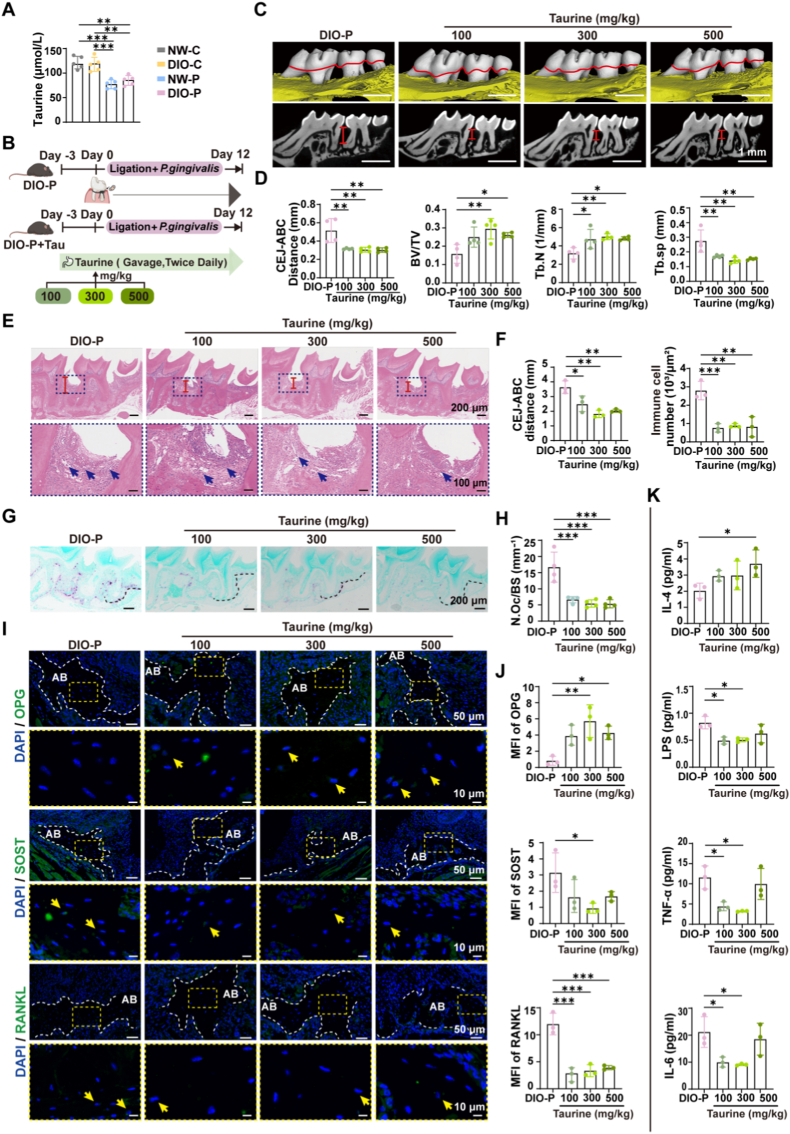
**Protective effects of taurine on periodontitis-induced bone damage in obese mice. A** Serum taurine levels in NW-C, DIO-C, NW-P, and DIO-P mice were measured using liquid chromatography‒tandem mass spectrometry (LC-MS/MS). **B** Schematic illustration of the experimental workflow. DIO-P mice received taurine supplements via combined oral gavage and drinking-water administration. Taurine was administered by oral gavage twice daily at 100, 300, or 500 mg/kg and simultaneously provided in the drinking water at 0.1%, 0.2%, or 0.4%, respectively. **C** Representative micro-CT images of maxillary alveolar bone shown as 3D reconstructions and sagittal views on day 12. Scale bar, 1 mm. **D** Quantitative micro-CT analysis of the CEJ–ABC distance, BV/TV, Tb.N, and Tb.Sp in the interdental region between the first and second molars. **E** Representative H&E-stained images. Red vertical line segments indicate the CEJ-ABC distance. The blue dashed boxes indicate enlarged gingival regions adjacent to the second molar, and the blue arrows indicate infiltrating immune cells. Scale bars, 200 μm and 100 μm. **F** Histomorphometric quantification of the CEJ–ABC distance and the number of infiltrating immune cells per unit area. **G** Representative images of TRAP-stained distal alveolar bone around the second molar. The black dashed lines demarcate the bone surface. Scale bar, 200 μm. **H** Quantification of TRAP-positive osteoclasts as N.Oc/BS in the distal alveolar bone of the second molar. **I** Representative images of IF staining showing OPG, SOST, and RANKL expression in the AB. Target proteins are presented in green, and nuclei are counterstained with DAPI (blue). Yellow dashed boxes mark enlarged bone regions, and yellow arrows indicate positive areas. White dashed lines demarcate bone boundaries. Scale bars, 50 μm and 10 μm. **J** Quantification analysis of the MFIs of OPG, SOST, and RANKL. **K** Circulating IL-4, LPS, TNF-α, and IL-6 levels detected using a Luminex multiplex assay. The data are shown as the means ± SDs. Differences among groups were analyzed using one-way ANOVA followed by Dunnett's multiple comparisons test. ∗*P* < 0.05, ∗∗*P* < 0.01, and ∗∗∗*P* < 0.001. *n* = 3–5 mice per group.

Given that taurine is an endogenous metabolite [39], we first evaluated systemic taurine availability by measuring serum taurine levels across groups. Compared with the NW-C, DIO-C, and NW-P mice, DIO-P mice showed significantly lower serum taurine levels (Fig. 5A), suggesting reduced systemic taurine availability in obese mice with periodontitis. Based on this finding, DIO-P mice were supplemented with taurine via a combination of oral gavage and the drinking water at concentrations of 100, 300, or 500 mg/kg by oral gavage and 0.1%, 0.2%, or 0.4% in the drinking water, respectively (Fig. 5B). Before evaluating its therapeutic effects, we first examined the systemic tolerability of taurine supplementation by performing multiorgan histological and serum biochemical analyses (Supplementary Fig. S5). H&E-stained sections from major organs, including the heart, liver, spleen, lung, kidney, and colon, showed no overt pathological abnormalities in DIO-P mice treated with any dose of taurine compared with those in untreated DIO-P mice (Supplementary Fig. S5A). Serum biochemistry revealed significantly reduced alanine aminotransferase (ALT) and d-lactate levels in all the taurine-treated groups, whereas the serum creatinine (CREA-S) level declined significantly only after 500 mg/kg taurine treatment; serum urea levels were not significantly altered among groups (Supplementary Fig. S5B). In addition, body weight was significantly reduced in mice treated with taurine at 300 and 500 mg/kg (Supplementary Fig. S5C). Overall, under the present experimental conditions, taurine supplementation was well tolerated by DIO-P mice. Having established its tolerability, we therefore proceeded to assess the impact of taurine supplementation on obesity-exacerbated alveolar bone loss. The micro-CT analysis showed that all tested doses of taurine supplementation markedly mitigated alveolar bone loss (Fig. 5C and D). Compared with untreated DIO-P mice, taurine-treated mice exhibited a shorter CEJ–ABC distance, higher BV/TV and Tb.N, and lower Tb.Sp (Fig. 5D), indicating improved preservation of the alveolar bone microarchitecture. The results of the histological analysis further supported these findings (Fig. 5E–H). H&E staining showed that compared with untreated DIO-P mice, taurine-treated DIO-P mice exhibited reduced periodontal tissue destruction and inflammatory cell infiltration, with a significantly shorter CEJ–ABC distance and fewer infiltrating immune cells (Fig. 5E and F). In parallel, TRAP staining and the histomorphometric analysis further demonstrated that taurine supplementation reduced osteoclast accumulation along the alveolar bone surface and significantly decreased N.Oc/BS in DIO-P mice (Fig. 5G and H). Together, these findings indicate that taurine administration at doses of 100, 300, and 500 mg/kg confers consistent protection against the obesity-induced exacerbation of alveolar bone destruction in DIO-P mice.

We further assessed whether taurine improves osteocyte-associated remodeling dysfunction in the obesity-associated periodontitis model by examining OPG, SOST, and RANKL expression in alveolar bone using IF staining. Compared with untreated DIO-P mice, 100, 300, and 500 mg/kg taurine supplementation reduced RANKL expression, treatment with 300 and 500 mg/kg taurine increased OPG expression, and treatment with 300 mg/kg taurine decreased SOST expression (Fig. 5I and J), suggesting a partial improvement in osteocyte-associated remodeling dysregulation in alveolar bone. We next assessed the impact of taurine supplementation on systemic inflammation by performing a multiplex analysis of serum samples. Compared with untreated DIO-P mice, treatment with 500 mg/kg taurine significantly increased IL-4 levels, whereas treatment with 100 and 300 mg/kg taurine significantly reduced the circulating levels of LPS, IL-6, and TNF-α (Fig. 5K). These findings indicate that taurine modulates systemic inflammatory alterations. Together, these findings show that taurine supplementation alleviated obesity-exacerbated alveolar bone damage, partially reversed the dysregulated osteocyte-associated remodeling, and modulated systemic inflammatory alterations in mice with periodontitis.

### 2.6 Taurine inhibits the obesity-exacerbated osteocyte ferroptosis in alveolar bone surrounding periodontitis-affected molars in mice

**Fig. 6:**
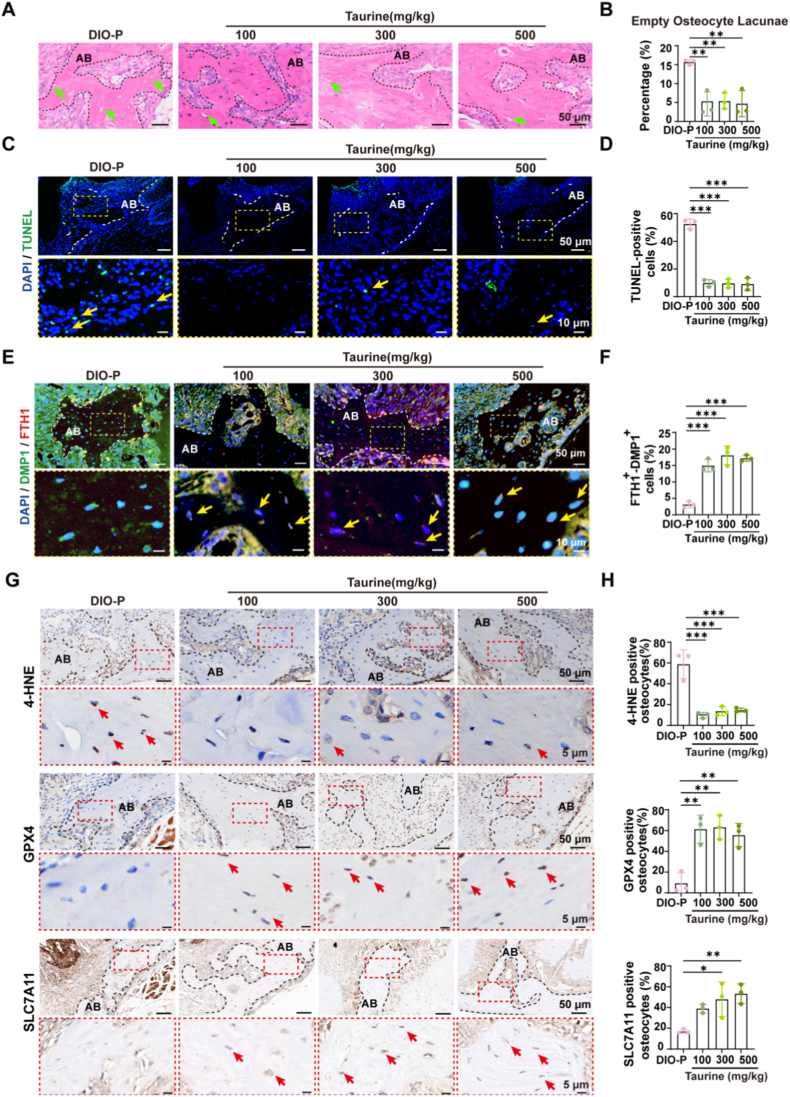
**Effects of taurine on osteocyte ferroptosis in the alveolar bone surrounding periodontitis-affected molars in obese mice. A** Representative H&E-staining images of alveolar bone sections from DIO-P mice treated with or without taurine. Green arrows indicate empty osteocyte lacunae, and the black dashed lines delineate the bone boundaries. Scale bar, 50 μm. **B** Histomorphometric quantification of the percentage of empty osteocyte lacunae in AB. **C** Representative fluorescence images of TUNEL-positive osteocytes (green) in AB. Nuclei were counterstained with DAPI (blue). Yellow dashed boxes indicate enlarged bone regions. Yellow arrows denote TUNEL-positive cells, and white dashed lines demarcate the bone boundaries. Scale bars, 50 μm and 10 μm. **D** Quantification of the percentage of TUNEL-positive osteocytes in AB. **E** Representative images of IF staining showing the colocalization of FTH1 (red) with DMP1 (green) in AB. Yellow dashed boxes indicate enlarged bone regions. Yellow arrows indicate FTH1^+^DMP1^+^cells, and white dashed lines demarcate the bone boundaries. Scale bars, 50 μm and 10 μm. **F** Quantitative assessment of the percentage of FTH1^+^DMP1^+^cells. **G** Representative images of IHC staining for 4-HNE, GPX4, and SLC7A11 in AB. Red dashed boxes indicate enlarged regions. Red arrows indicate positively stained osteocytes, and black dashed lines delineate the bone boundaries. Scale bars, 50 μm and 5 μm. **H** Quantification of the percentages of 4-HNE-, GPX4-, and SLC7A11-positive osteocytes in AB. The data are shown as the means ± SDs. Differences among groups were analyzed using one-way ANOVA followed by Dunnett's multiple comparisons test. ∗*P* < 0.05, ∗∗*P* < 0.01, and ∗∗∗*P* < 0.001. *n* = 3 mice per group.

We evaluated osteocyte injury and ferroptosis-associated markers in alveolar bone from DIO-P mice treated with 100, 300, or 500 mg/kg taurine to determine whether taurine-mediated bone protection was associated with reduced osteocyte ferroptosis *in vivo*. Histological and quantitative analyses showed that, compared with untreated DIO-P mice, all of the tested doses of taurine reduced the percentage of empty osteocyte lacunae (Fig. 6A and B). Similarly, TUNEL staining and quantification revealed that treatment with 100, 300, and 500 mg/kg taurine significantly decreased the proportion of TUNEL-positive osteocytes in alveolar bone (Fig. 6C and D), indicating reduced osteocyte death following taurine treatment. We next evaluated FTH1 expression in osteocytes by performing dual immunofluorescence staining for FTH1 and DMP1. Compared with untreated DIO-P mice, all tested doses of taurine increased the proportion of FTH1^+^DMP1^+^ cells (Fig. 6E and F), suggesting that the iron storage capacity of osteocytes was partially restored following taurine treatment. Consistent with these findings, the IHC analysis showed that treatment with 100, 300, and 500 mg/kg taurine reduced 4-HNE staining and increased GPX4 expression, whereas treatment with 300 and 500 mg/kg taurine significantly increased SLC7A11 expression compared with untreated DIO-P mice (Fig. 6G and H). Collectively, these data indicate that taurine attenuates the obesity-induced aggravation of osteocyte ferroptosis in alveolar bone during periodontitis, as reflected by improved osteocyte preservation, reduced lipid peroxidation, and the restoration of antiferroptotic defense pathways.

### 2.7 Activation of ferroptosis attenuates the protective effects of taurine on obesity-exacerbated periodontitis

**Fig. 7:**
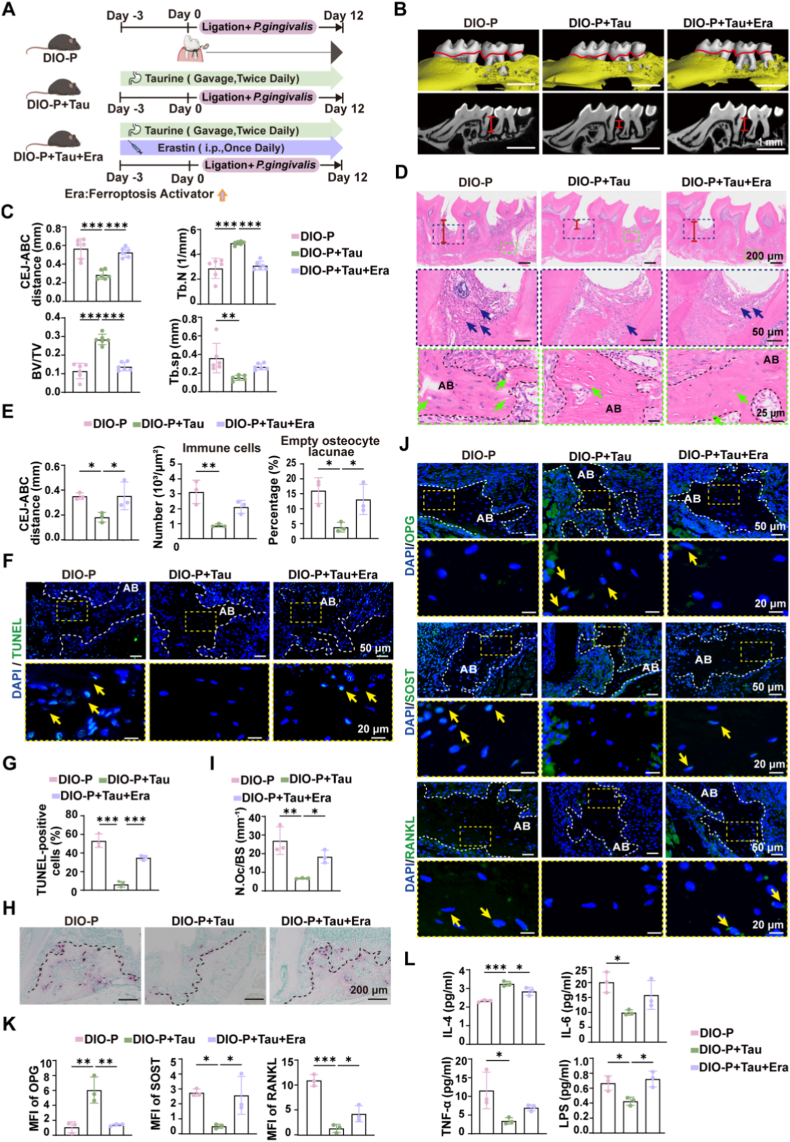
**Effects of ferroptosis activation on bone damage in taurine-treated obese mice with periodontitis. A** Schematic illustration of the experimental workflow. Based on the preceding dose-screening study, 300 mg/kg taurine was selected for subsequent mechanistic experiments. Three groups were included: untreated DIO-P mice (DIO-P), taurine-treated DIO-P mice (DIO-P + Tau), and taurine-treated DIO-P mice co-administered with the ferroptosis activator erastin (Era) (DIO-P + Tau + Era). Taurine was administered by oral gavage (300 mg/kg, twice daily) together with taurine-supplemented drinking water (0.2%), whereas erastin was administered intraperitoneally at 40 mg/kg once daily. **B** Representative micro-CT scans of maxillary alveolar bone shown as 3D reconstructions and sagittal views on day 12. Red lines mark the CEJ, and red line segments indicate the CEJ–ABC distance. Scale bar, 1 mm. **C** Quantitative micro-CT analysis of the CEJ–ABC distance, BV/TV, Tb.N and Tb.Sp in the interdental region between the first and second molars. **D** Representative H&E-stained images. Red vertical line segments indicate the CEJ–ABC distance. The blue dashed boxes denote enlarged images of the mesial gingiva adjacent to the second molar, and the green dashed boxes denote enlarged images of the distal alveolar bone adjacent to the second molar. The blue arrows indicate infiltrating immune cells, and the green arrows indicate empty osteocyte lacunae. Black dashed lines outline the bone boundaries. Scale bars, 200 μm (top panels), 50 μm (middle panels), and 25 μm (bottom panels). **E** Histomorphometric quantification of the CEJ–ABC distance, the number of infiltrating immune cells per unit area, and the percentage of empty osteocyte lacunae. **F** Representative fluorescence images of TUNEL-positive osteocytes (green) in AB. Nuclei were counterstained with DAPI (blue). The white dashed lines demarcate bone boundaries, and the yellow arrows denote TUNEL-positive cells. Scale bars, 50 μm and 20 μm. **G** Quantitative assessment of the percentage of TUNEL-positive osteocytes in AB. **H** Representative images of TRAP-stained distal alveolar bone around the second molar. The black dashed lines delineate the bone surface. Scale bar, 200 μm. **I** Quantification of TRAP-positive osteoclasts expressed as N.Oc/BS. **J** Representative images of IF staining for OPG, SOST, and RANKL in AB. Target proteins are presented in green, and nuclei are counterstained with DAPI (blue). The yellow dashed boxes indicate enlarged bone regions. Yellow arrows indicate positively stained areas, and the white dashed lines demarcate the bone boundaries. Scale bars, 50 μm and 20 μm. **K** Quantification analysis of the MFIs of OPG, SOST, and RANKL. **L** Circulating IL-4, LPS, TNF-α, and IL-6 levels detected using a Luminex multiplex assay. The data are shown as the means ± SDs. Differences among groups were analyzed using one-way ANOVA followed by Dunnett's multiple comparisons test. ∗*P* < 0.05, ∗∗*P* < 0.01, and ∗∗∗*P* < 0.001. *n* = 3–6 mice per group.

Having shown that taurine supplementation alleviates the obesity-induced aggravation of periodontal bone damage, attenuates systemic inflammatory burden, and reduces ferroptosis-associated osteocyte injury *in vivo*, we next sought to determine whether these protective effects were mediated, at least in part, by the suppression of osteocyte ferroptosis. Based on the preceding dose-response results, 300 mg/kg taurine was selected as an intermediate effective dose that produced robust and consistent protective effects across the evaluated outcomes for subsequent mechanistic experiments. DIO-P mice treated with taurine were coadministered erastin, a well-established ferroptosis inducer, to generate the DIO-P + Tau + Era group, while untreated DIO-P mice (DIO-P) and taurine-treated DIO-P mice (DIO-P + Tau) served as control groups (Fig. 7A). IF and IHC staining were performed to assess the expression of ferroptosis-related markers and to determine whether erastin could reverse the suppressive effect of taurine on osteocyte ferroptosis in alveolar bone. Compared with the DIO-P + Tau mice, the DIO-P + Tau + Era mice presented a reduced proportion of FTH1^+^DMP1^+^ cells, increased 4-HNE accumulation, and decreased GPX4 and SLC7A11 expression (Supplementary Fig. S6), indicating that erastin effectively reversed the taurine-mediated suppression of osteocyte ferroptosis. The micro-CT analysis showed that erastin cotreatment partially reversed the protective effects of taurine on alveolar bone and the trabecular microarchitecture. Compared with DIO-P + Tau mice, DIO-P + Tau + Era mice presented a significantly greater CEJ–ABC distance, lower BV/TV and Tb.N, and greater Tb.Sp (Fig. 7B and C), indicating that the reactivation of ferroptosis compromises taurine-mediated bone protection. H&E staining and the histomorphometric analysis further revealed a greater CEJ–ABC distance and a higher percentage of empty osteocyte lacunae in DIO-P + Tau + Era mice than in DIO-P + Tau mice. These findings suggested that erastin cotreatment attenuated the taurine-mediated preservation of periodontal architecture and osteocyte integrity (Fig. 7D and E). Consistent with these findings, TUNEL staining showed a significantly higher proportion of TUNEL-positive osteocytes in DIO-P + Tau + Era mice than in DIO-P + Tau mice (Fig. 7F and G), further supporting increased osteocyte death following ferroptosis reactivation. TRAP staining and the histomorphometric analysis further showed increased osteoclast accumulation along the alveolar bone surface and a significantly higher N.Oc/BS in DIO-P + Tau + Era mice than in DIO-P + Tau mice (Fig. 7H and I).

We next assessed whether erastin cotreatment reversed the restoration of osteocyte-derived remodeling signals induced by taurine. IF staining showed that, compared with DIO-P + Tau mice, DIO-P + Tau + Era mice presented lower OPG expression and higher SOST and RANKL expression in alveolar bone (Fig. 7J and K), indicating that ferroptosis reactivation shifted the osteocyte-derived remodeling balance toward a proresorptive state despite taurine treatment. We further assessed the serum levels of circulating inflammatory markers by performing a Luminex analysis to determine whether ferroptosis reactivation also affected the systemic inflammatory profile in taurine-treated obese mice with periodontitis. Compared with DIO-P + Tau mice, DIO-P + Tau + Era mice presented reduced circulating IL-4 levels and increased LPS levels (Fig. 7L), indicating that ferroptosis reactivation partially attenuated the systemic anti-inflammatory effects of taurine. Collectively, these findings indicate that the reactivation of ferroptosis partially reverses the protective effects of taurine on obese mice with periodontitis, suggesting that the suppression of osteocyte ferroptosis is an important mechanism underlying taurine-mediated protection.

### 2.8 The activation of ferroptosis attenuates the protective effects of taurine on osteocyte function upon PA and LPS exposure

**Fig. 8:**
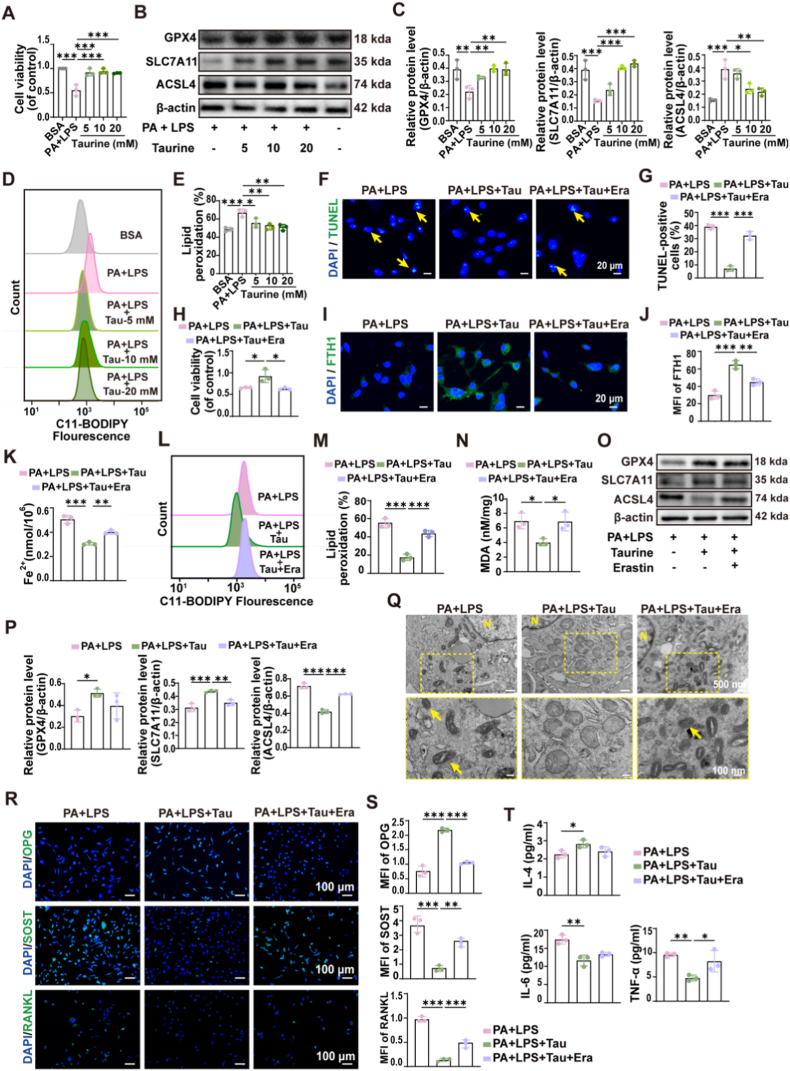
The role of ferroptosis in the taurine-mediated restoration of osteocyte function and cytokine production in MLO-Y4 osteocytes upon PA and LPS exposure. **A** Viability of MLO-Y4 cells treated with BSA (control) or PA (100 μM) plus LPS (1 μg/mL) in the presence or absence of taurine (5, 10, or 20 mM) for 24 h. **B** Representative western blots showing GPX4, SLC7A11, and ACSL4 expression in MLO-Y4 cells. **C** Densitometric quantification of GPX4, SLC7A11, and ACSL4 protein expression after normalization to β-actin. **D** Representative flow cytometry plots of lipid ROS generation detected using C11-BODIPY staining. **E** Quantification of lipid peroxidation, expressed as the percentage of C11-BODIPY-positive cells. **F–T** Based on the dose screening results shown in A–E, 10 mM taurine was selected for subsequent mechanistic experiments. MLO-Y4 cells exposed to PA (100 μM) and LPS (1 μg/mL) were divided into the following three groups to further assess the involvement of ferroptosis in taurine-mediated protection: PA + LPS, PA + LPS + Tau (10 mM), and PA + LPS + Tau (10 mM) + Era (10 μM). **F** Representative fluorescence images of TUNEL (green) staining. Nuclei were counterstained with DAPI (blue). Yellow arrows indicate TUNEL-positive cells. Scale bar, 20 μm. **G** Quantitative assessment of TUNEL-positive cells. **H** Cell viability determined by CCK-8 assay. **I** Representative images of IF staining for FTH1 (green) in MLO-Y4 cells. Nuclei were counterstained with DAPI (blue). Scale bar, 20 μm. **J** MFI analysis of FTH1. **K** Intracellular Fe^2+^ content determined using a colorimetric assay and normalized to cell number (nmol/10^6^ cells). **L** Representative flow cytometry plots of lipid ROS generation detected by C11-BODIPY staining in response to erastin cotreatment. **M** Quantitative analysis of lipid peroxidation. **N** MDA levels were measured by performing a colorimetric assay and normalized to the total protein content (nmol/mg). **O** Representative western blots showing GPX4, SLC7A11, and ACSL4 expression. **P** Densitometric quantification of GPX4, SLC7A11, and ACSL4 protein expression after normalization to β-actin. **Q** Representative TEM images of the perinuclear region showing mitochondrial and nuclear ultrastructural changes. Yellow dashed boxes indicate enlarged views highlighting the mitochondrial ultrastructure, and yellow arrows indicate characteristic ferroptosis-like changes. Scale bars, 500 nm and 100 nm. **R** Representative images of IF staining for OPG, SOST, and RANKL in MLO-Y4 cells. The target proteins are presented in green, and nuclei are counterstained with DAPI (blue). Scale bar, 100 μm. **S** Quantification analysis of the MFIs of OPG, SOST, and RANKL. **T** IL-4, IL-6, and TNF-α levels in culture supernatants detected by a Luminex multiplex assay. The data are shown as the means ± SDs. Differences among groups were analyzed using one-way ANOVA followed by Dunnett's multiple comparisons test. ∗*P* < 0.05, ∗∗*P* < 0.01, and ∗∗∗*P* < 0.001. *n* = 3 independent experiments.

MLO-Y4 cells were treated with PA + LPS in the presence of 5, 10, or 20 mM taurine to determine the optimal taurine concentration for rescuing PA + LPS-induced osteocyte injury. All tested concentrations of taurine significantly increased cell viability, with 10 mM exerting the strongest protective effect (Fig. 8A). In parallel, taurine partially reversed the PA + LPS-triggered changes in ferroptosis-associated protein expression. In particular, 10 mM taurine markedly enhanced GPX4 and SLC7A11 expression but suppressed ACSL4 levels (Fig. 8B and C). Consistent with these findings, taurine reduced PA + LPS-induced lipid peroxidation, with 10 mM taurine providing substantial suppression (Fig. 8D and E). Therefore, 10 mM taurine was selected for subsequent mechanistic experiments. Ferroptosis was pharmacologically reactivated using erastin to determine whether taurine-mediated protection depends on ferroptosis inhibition. Compared with taurine treatment alone, erastin cotreatment increased the proportion of TUNEL-positive cells and significantly blunted the taurine-mediated recovery of cell viability (Fig. 8F–H). In parallel, erastin cotreatment partially abolished the taurine-induced improvement in iron homeostasis, as evidenced by lower FTH1 expression and higher intracellular labile Fe^2+^ levels in the erastin-treated group than in the taurine-treated group (Fig. 8I–K). Moreover, compared with taurine treatment alone, erastin cotreatment restored lipid ROS accumulation and increased MDA levels (Fig. 8L–N). Consistent with these findings, erastin cotreatment partially reversed taurine-induced upregulation of GPX4 and SLC7A11 and downregulation of ACSL4 (Fig. 8O and P). TEM analysis further showed that, compared with taurine treatment alone, erastin cotreatment led to the reappearance of ferroptosis-like mitochondrial abnormalities, including mitochondrial shrinkage, an increased membrane electron density, and disrupted cristae (Fig. 8Q). Collectively, these findings suggest that ferroptosis reactivation partially compromises the taurine-mediated suppression of osteocyte ferroptosis under PA + LPS stimulation. We next investigated whether erastin could also weaken the beneficial effects of taurine on osteocyte-derived bone remodeling signals and inflammatory cytokine production. IF staining showed that, compared with taurine treatment alone, erastin cotreatment reduced OPG expression and increased SOST and RANKL signals (Fig. 8R and S), indicating a partial loss of taurine-mediated protection against the proresorptive shift in the osteocyte phenotype. In addition, erastin cotreatment partially reversed the suppressive effect of taurine on TNF-α secretion (Fig. 8T). Taken together, these results indicate that taurine mitigates PA + LPS-induced osteocyte dysfunction and inflammatory burden, whereas the pharmacological reactivation of ferroptosis partially abolishes these beneficial effects, highlighting ferroptosis inhibition as an important mechanism underlying taurine-mediated osteocyte protection.

## 3 Discussion

Epidemiological studies have consistently shown that obesity is associated with an increased risk and greater severity of periodontitis, accompanied by more extensive alveolar bone destruction [[40], [41], [42]]. However, the mechanisms by which obesity exacerbates periodontal bone loss remain incompletely understood. Osteocytes, the most abundant type of bone-resident cell, serve not only as mechanosensors but also as central regulators of bone remodeling, and their dysfunction can promote osteoclastogenesis and pathological bone resorption [43]. Recent evidence has implicated osteocyte ferroptosis in alveolar bone loss during periodontitis [23], but its role in obesity-associated periodontitis has not yet been defined. In the present study, we showed that obesity aggravated periodontitis-associated alveolar bone loss by increasing osteocyte ferroptosis, and that taurine markedly attenuated these pathological changes. Together, these findings identify osteocyte ferroptosis as a mechanistically important link between obesity and aggravated periodontal bone destruction and suggest that targeting this pathway may represent a therapeutic strategy for obesity-associated periodontitis.

Our *in vivo* data revealed that obesity markedly exacerbated periodontitis-induced alveolar bone loss, inflammatory cell infiltration, and osteoclast accumulation (Fig. 1), consistent with the view that obesity creates a proinflammatory and metabolically dysregulated milieu that amplifies periodontal tissue injury [[44], [45], [46]]. Among the mediators examined, TNF-α levels in serum and RANKL levels in alveolar bone were selectively increased under obese conditions, supporting the hypothesis that a systemic metabolic disturbance is coupled to a more proresorptive periodontal bone microenvironment [10,47].

A central finding of this study is that osteocyte ferroptosis functionally contributes to the obesity-exacerbated periodontal bone loss. This conclusion is supported by a stepwise line of evidence: the transcriptomic analysis first revealed the selective enrichment of ferroptosis-related signatures under the combined condition of obesity and periodontitis (Fig. 2), histological and molecular assays confirmed increased ferroptotic injury in alveolar osteocytes (Fig. 2, Fig. 4), and pharmacological inhibition with Fer-1 functionally attenuated obesity-exacerbated alveolar bone loss (Fig. 3). A plausible explanation for this effect is that obesity and periodontitis together create a microenvironment that is particularly permissive for ferroptosis. While periodontitis induces sustained inflammatory stress [5], obesity contributes to chronic lipid excess, redox imbalance, and disturbed iron homeostasis [48,49], all of which are well-recognized drivers of ferroptotic vulnerability [50]. Under these combined conditions, alveolar bone cells are likely exposed not only to intensified inflammation but also to a metabolic milieu that favors lipid peroxidation and weakens antioxidant defenses, thereby lowering the threshold for ferroptosis.

These findings also support the emerging concept that osteocyte ferroptosis is a shared mechanism linking systemic metabolic stress to local bone damage. Osteocytes are long-lived, matrix-embedded cells with limited regenerative capacity and play a central role in coordinating bone remodeling [43,51], making them especially susceptible to persistent oxidative and metabolic stress. Consistent with this view, osteocyte ferroptosis has been implicated in several pathological bone-loss conditions, including diabetic osteoporosis, postmenopausal osteoporosis, and diabetic periodontitis [[52], [53], [54]]. Our results extend this framework to obesity-associated periodontitis and further support the hypothesis that the preservation of osteocyte viability may help maintain remodeling homeostasis under pathological metabolic stress.

Taurine has emerged as a rational candidate because it is tightly linked to redox and lipid homeostasis—core processes governing ferroptosis susceptibility [[55], [56], [57]]. Notably, we observed a significant reduction in circulating taurine levels in DIO-P mice (Fig. 5A), consistent with recent clinical evidence showing lower taurine levels in periodontitis and an inverse association with disease severity [58,59]. These findings suggest that taurine depletion may be part of the metabolic imbalance associated with obesity-associated periodontitis and provide a rationale for testing taurine supplementation as a metabolically informed intervention. Functionally, taurine supplementation conferred broad protection *in vivo*, including the attenuation of alveolar bone loss, reduced osteoclast accumulation, partial correction of osteocyte-mediated remodeling imbalance, and modulation of systemic inflammatory alterations (Fig. 5). These effects are consistent with previous evidence that taurine supports bone metabolism and skeletal homeostasis through antioxidant, anti-inflammatory, and metabolic regulatory actions [28,60]. While previous studies have focused mainly on osteoblasts and osteoclasts [61], the role of taurine in osteocytes, key regulators of bone remodeling and skeletal homeostasis [62,63], remains unclear. Therefore, our results broaden current knowledge by demonstrating that taurine preserves osteocyte viability and function during obesity-exacerbated periodontitis.

Mechanistically, our data support the idea that the suppression of osteocyte ferroptosis is an important mediator of the effects of taurine. Taurine consistently ameliorated ferroptosis-associated changes in both alveolar bone and osteocyte cultures (Fig. 6, Fig. 8), whereas the pharmacological reactivation of ferroptosis with erastin partially blunted these protective effects (Fig. 7, Fig. 8). This incomplete reversal by erastin suggests that ferroptosis inhibition is not the sole mechanism underlying taurine-mediated protection. Taurine has also been implicated in the preservation of mitochondrial function and the rescue of mitochondria-related metabolic impairments [[64], [65], [66]], the modulation of endoplasmic reticulum stress [67], and the maintenance of pro-survival signaling in bone-related cells [60,62], all of which could influence osteocyte stress tolerance and remodeling capacity. Taurine-mediated bone protection in this model is therefore likely multifactorial, with the suppression of ferroptosis representing a major, but not exclusive, component.

This broader mode of action enhances the translational relevance of taurine. In the context of obesity-associated periodontitis, targeting osteocyte ferroptotic vulnerability may represent a promising strategy for limiting periodontal bone destruction. More broadly, because ferroptosis has also been implicated in other disorders of skeletal homeostasis [17,68], taurine may have therapeutic potential beyond its role in treating periodontal disease under pathological conditions characterized by metabolic stress and bone loss. Taken together, these findings support the use of taurine as a biologically plausible candidate for further investigations of ferroptosis-related skeletal disorders.

Our data also suggest that taurine acts in a nonlinear dose-dependent manner (Fig. 5). Low doses were sufficient to confer bone protection, whereas higher doses provided limited additional benefit, suggesting an early plateau in local bone responses. One possible explanation is saturable taurine uptake or tissue distribution, which could limit further increases in local bioavailability despite increased systemic exposure [69,70]. In addition, given the role of taurine in osmotic regulation and membrane stabilization, excessive exposure may induce adaptive responses that attenuate incremental benefits [56,71]. Its immunomodulatory effects may also be context- and pathway-dependent rather than uniformly suppressive [[72], [73], [74]], which is consistent with our observation that higher doses did not result in proportionally greater reductions in proinflammatory cytokine levels and were instead associated with increased IL-4 levels. Collectively, these findings point to the existence of an optimal therapeutic window for taurine, which warrants further definition in future studies.

Several limitations should be noted. Our conclusions were derived mainly from a murine model and complementary *in vitro* experiments; therefore, confirmation in humans with obesity-associated periodontitis is needed. In particular, whether reduced taurine levels and increased osteocyte ferroptosis occur in patients, and whether these changes correlate with periodontal severity and the metabolic status, remain unknown. In addition, the upstream mechanism of systemic taurine deficiency and its causal links to ferroptosis activation, osteocyte dysfunction, and alveolar bone loss have not been fully elucidated. Finally, because taurine exhibited a nonlinear dose profile in our study, future work will be needed to define its optimal therapeutic window.

## 4 Conclusions

Our findings confirmed that obesity aggravates periodontitis-associated alveolar bone destruction and is accompanied by increased osteocyte ferroptosis in alveolar bone. Osteocyte ferroptosis represents a key mechanism underlying this pathological interaction. Taurine alleviates the obesity-induced aggravation of periodontal bone loss, at least in part, by suppressing osteocyte ferroptosis. Given the lack of targeted therapies for periodontitis complicated by systemic metabolic disorders, our findings identify osteocyte ferroptosis as a promising therapeutic target and suggest taurine as a potential intervention for complex periodontitis associated with obesity and related metabolic dysfunction.

## 5 Materials and methods

### 5.1 Animals and experimental design

Male C57BL/6J mice at the age of 5–6 weeks were purchased from Wuhan Shouzheng Hongyao Biotechnology Co., Ltd. (Wuhan, China; license No. SCXK (Hubei) 2023-0034). After a 1-week acclimation period, the mice were randomly allocated to either a normal diet group or a diet-induced obesity (DIO) group. The mice in the DIO group were fed a 60% high-fat diet (MD12033, Medison) for 12 weeks to establish the obesity model [75,76], whereas the mice in the normal diet group were maintained on a standard chow diet and served as the normal-weight (NW) group. All mice were kept in a controlled environment with a 12-h light/12-h dark cycle, with ad libitum access to food and water. Body weight was measured weekly throughout the 12-week feeding period, while blood glucose levels were detected at weeks 0, 8, and 12. At week 12, the successful establishment of the DIO model was confirmed via oral glucose tolerance test (OGTT) and body composition analysis. After the obesity model was established, NW and DIO mice were further randomly assigned to a control subgroup (C) and a periodontitis subgroup (P).

To investigate the effect of obesity on periodontitis-associated alveolar bone destruction and osteocyte injury, four experimental groups were established: NW-C (normal-weight control), NW-P (normal-weight with periodontitis), DIO-C (diet-induced obese control), and DIO-P (diet-induced obese with periodontitis).

For mice in the periodontitis subgroups, experimental periodontitis was induced by placing a 5–0 silk ligature around both maxillary second molars, combined with topical administration of *P. gingivalis* [[32], [33], [34]]. A 20-μL aliquot of the prepared bacterial suspension (preparation details are described in Section 5.4) was carefully delivered to the gingival sulcus and ligature around the maxillary second molars using a microsyringe. To further prevent physical washout, the mice were restrained from food and water intake for 1 h immediately after each inoculation [77]. This topical application was performed three times weekly over a 12-day period [78].

For *in vivo* pharmacological intervention, treatments were initiated 3 days before ligature placement and *P. gingivalis* application and continued throughout the experimental period. Because taurine (Tau; Sigma‒Aldrich, T0625) has a relatively short half-life, a combined oral gavage plus drinking water supplementation regimen was used to maintain more stable systemic exposure. Taurine was administered twice daily by oral gavage at 100, 300, or 500 mg/kg and was simultaneously provided in the drinking water at 0.1%, 0.2%, or 0.4% (weight/volume; equivalent to 1, 2, or 4 mg/mL) for the low-, medium-, and high-dose groups, respectively [[79], [80], [81]]. Based on the estimated body weight and daily water intake of the mice, these concentrations in the drinking water were used as practical maintenance doses. For ferroptosis-targeted interventions, the ferroptosis inhibitor ferrostatin-1 (Fer-1; Selleck, S7243) was administered by intraperitoneal injection at 10 mg/kg once daily, and the ferroptosis activator erastin (Era; Selleck, S7242) was administered intraperitoneally at 40 mg/kg once daily. All animal procedures were approved by the Institutional Animal Care and Use Committee of the Fourth Military Medical University (Ethical Approval Number: 20260060) and were performed in strict compliance with the NIH Guide for the Care and Use of Laboratory Animals. At the end of the experiment, the mice were subjected to deep anesthesia with isoflurane and euthanized by cervical dislocation.

### 5.2 Blood glucose measurement and oral glucose tolerance test (OGTT)

Random blood glucose levels were detected in the morning at weeks 0, 8, and 12 during the feeding period. Blood samples were collected from the tail vein, and glucose levels were determined using an Accu-Chek glucometer with the corresponding test strips (Roche Diagnostics, Basel, Switzerland).

For the OGTT assay, these mice were fasted for 12–16 h prior to the test, followed by oral gavage of d-glucose at a dose of 2 g/kg body weight (Orileaf, A10014). Blood glucose levels were measured in tail vein blood samples at 0, 15, 30, 45, and 60 min using an Accu-Chek glucometer and corresponding test strips (Roche Diagnostics). The area under the curve (AUC) was calculated to assess the glucose tolerance of the mice [82].

### 5.3 Body composition analysis

At the end of the 12-week dietary intervention, body composition was assessed in NW and DIO mice using a Bruker LF90 small-animal body composition analyzer (Bruker, Germany) according to a previously described protocol [83]. Parameters including fat mass and body fat percentage were recorded and used to further confirm the successful establishment of the DIO model.

### 5.4 Preparation of *P. gingivalis* suspension

*P. gingivalis* (ATCC 33277) was purchased from BIO SCI Biotechnology (Zhejiang, China). The strain was cultured in sterile brain heart infusion (BHI) broth (Hopebiol, HB8297-1) supplemented with 5 μg/mL hemin (Hopebiol) and 1 μg/mL vitamin K1 (Hopebiol). Incubation was performed at 37 °C under strict anaerobic atmosphere composed of 80% N_2_, 10% H_2_ and 10% CO_2_ sustained by a GasPak anaerobic system (AN0025A, Thermo Scientific, CA, USA) for 24–48 h [84,85]. Bacterial growth was monitored by measuring the optical density at 600 nm (OD_600_) with a turbidimeter (BD PhoenixSpec™), and bacteria were harvested at the mid-logarithmic phase when the OD_600_ reached 0.5–0.7 [86,87]. The cultured bacterial suspension was collected via centrifugation at 6000 *g* for 10 min at 4 °C. Bacteria were washed in PBS and prepared at a final concentration of 1 × 10^9^ colony-forming units (CFU)/mL in sterile PBS containing 2% (weight/volume) carboxymethyl cellulose (CMC; Sigma Aldrich, 045847-22) [88,89]. All prepared bacterial suspensions were temporarily stored at 4 °C prior to topical application.

### 5.5 Micro-CT analysis

Fixed maxillary samples were scanned with a high-resolution micro-CT scanner (AX2000 CT, Always Imaging Co., Ltd., Shanghai, China). VG Studio MAX 3.5.1 (Volume Graphics GmbH, Heidelberg, Germany) was used for three-dimensional (3D) model reconstruction and quantitative analysis. Alveolar bone resorption was assessed by measuring the linear distance between the cementoenamel junction (CEJ) and the alveolar bone crest (ABC) at the mesial site of the ligated maxillary second molar. A standardized region of interest (ROI) was defined in the interdental alveolar bone between the first and second molars to quantify trabecular microarchitectural parameters, including the bone volume/tissue volume (BV/TV), trabecular number (Tb.N), and trabecular separation (Tb.Sp) [90].

### 5.6 Histological and histomorphometric analyses

Decalcified maxillary sections (4 μm) were subjected to deparaffinization and rehydration, followed by hematoxylin and eosin staining (H&E; BIOSSCI Biotech, BP0211) for histomorphological observation. Osteoclast detection was performed using a Tartrate-Resistant Acid Phosphatase (TRAP) Staining Kit (Sigma-Aldrich, 387A-1 KT) following previously established methods [91], with methyl green used for nuclear counterstaining. The degree of alveolar bone loss was determined by measuring the CEJ–ABC linear distance at the mesial side of the maxillary second molar. Immune cell infiltration within gingival connective tissue was quantified and reported as the number of immune cells per unit area (cells/μm^2^). Empty osteocyte lacunae were defined as lacunar spaces lacking a visible nucleus or cytoplasmic profile [23]. The percentage of empty lacunae was calculated relative to the total number of lacunae within the alveolar bone matrix. TRAP-positive osteoclasts adjacent to the second molar were quantified as osteoclast number per bone surface (N.Oc/BS), a reliable parameter for evaluating osteoclastogenesis in the periodontitis microenvironment [92].

### 5.7 Immunohistochemical (IHC) and immunofluorescence (IF) staining

IHC staining was applied to assess the expression levels of ferroptosis-related proteins [93], such as 4-hydroxynonenal (4-HNE), glutathione peroxidase 4 (GPX4), and solute carrier family 7 member 11 (xCT/SLC7A11), in alveolar bone. The primary antibodies and dilutions used in this experiment were as follows: 4-HNE (Bioss, bs-6313R; 1:50), GPX4 (Proteintech, 30388-1-AP; 1:100), and SLC7A11 (Proteintech, 32384-1-AP; 1:50).

IF staining was performed to evaluate the expression of markers related to bone homeostasis, including OPG, SOST, and RANKL. Sections were costained with antibodies against FTH1 and the osteocyte marker dentin matrix protein 1 (DMP1) to assess changes in iron handling associated with osteocyte ferroptosis [22]. The primary antibodies and dilutions used in this experiment were as follows: OPG (Proteintech, 30870-1-AP; 1:200), SOST (Proteintech, 21933-1-AP; 1:100), RANKL (Affinity, AF0313; 1:100), FTH1 (Abcam, ab75973; 1:150), and DMP1 (Bioss, bs-25502R; 1:200). DAPI (Solarbio, C0060) was used for nuclear counterstaining.

For cellular IF staining, cells were fixed with 4% paraformaldehyde (Beyotime, P0099) for 30 min, washed with PBS, permeabilized with 0.1% Triton X-100 (Beyotime, P0096) for 10 min, and washed again with PBS. After blocking with immunostaining blocking buffer (Beyotime, P0104) for 60 min, cells were incubated at 4 °C for 16 h with primary antibodies diluted in QuickBlock™ primary antibody dilution buffer (Beyotime, P0263): RANKL (Affinity, AF0313; 1:300); SOST (Proteintech, 21933-1-AP; 1:300); OPG (Proteintech, 11534-1-AP; 1:300); and FTH1 (Abcam, ab75973; 1:300). After washes with PBS, cells were incubated at room temperature for 1 h with Alexa Fluor 488-conjugated donkey anti-rabbit IgG secondary antibody (Abbkine, A23220; 1:500) diluted in QuickBlock™ secondary antibody dilution buffer (Beyotime, P0265). Cell nuclei were counterstained using DAPI (Solarbio). Images were acquired with a confocal microscope (Nikon, Tokyo, Japan) and signals were quantified using Fiji software (ImageJ v1.54p, NIH, Bethesda, MD, USA).

### 5.8 Image acquisition and quantitative analysis

All sections subjected to histological, IHC, and IF staining were digitized using a high-resolution slide scanner (VS200, Olympus, Tokyo, Japan). Quantitative analyses were performed using OlyVIA software (v3.4.1, Evident Scientific, Tokyo, Japan) for regional measurements and Fiji (NIH) for signal quantification.

### 5.9 Luminex multiplex assay

Concentrations of interleukin-6 (IL-6), tumor necrosis factor-α (TNF-α) and interleukin-4 (IL-4) in serum and cell culture supernatants were measured using a Luminex-based multiplex assay kit (USCN Life Science Inc., Wuhan, China) according to the manufacturer's instructions. Measurements were performed on a Luminex 200 system (Luminex Corp., Austin, TX, USA). The serum lipopolysaccharide (LPS) level was measured with the same platform using the corresponding assay provided in the kit series. The concentrations were calculated based on the median fluorescence intensity (MFI) and standard curves.

### 5.10 Liquid chromatography‒tandem mass spectrometry (LC-MS/MS)

Serum taurine content was determined via LC-MS/MS analysis. Serum specimens underwent extraction using a mixed solution of methanol, acetonitrile and water, followed by centrifugation, drying, and redissolution prior to analysis. Chromatographic separation was performed on a Shimadzu liquid chromatography system (Shimadzu, Kyoto, Japan) with a Restek Allure® PFP Propyl column (100 mm × 2.1 mm, 5 μm; Restek, Bellefonte, PA, USA), and mass spectrometric detection was performed on an API 4000 LC/MS/MS system (AB Sciex, Framingham, MA, USA) in positive ion multiple reaction monitoring mode. Taurine concentrations were determined using isotope-labeled internal standards and calibration curves [94].

### 5.11 RNA sequencing (RNA-seq) profiling

Adherent soft tissues were gently removed from the maxillary bone surrounding the ligated molars to eliminate transcriptomic noise from gingival tissues. Total RNA was subsequently isolated from alveolar bone samples of the NW-C, NW-P, DIO-C, and DIO-P groups (n = 5) using the phenol–guanidine isothiocyanate method. The RNA concentration and integrity were validated using a Qubit 4.0 fluorometer (Thermo Fisher Scientific, Waltham, MA, USA) and a Qsep 400 biofragment analyzer (BiOptic Inc., New Taipei City, Taiwan, China), respectively. The cDNA libraries were constructed and sequenced by MetWare Biotechnology Co., Ltd. (Wuhan, China) on the DNBSEQ-T7 platform (MGI Tech, Shenzhen, China). Raw sequencing data were filtered by Fastp (version 0.23.2) and then mapped to the mouse reference genome (GRCm39.109) via HISAT2 (version 2.2.1). Gene read counts were generated with featureCounts (version 2.0.3). Normalized transcript abundance presented as fragments per kilobase of transcript per million mapped reads (FPKM) was applied for data visualization, whereas raw count data were imported into DESeq2 (version 1.38.3) for differential expression screening. Genes with P < 0.05 and |log2FC| > 0.585 were regarded as differentially expressed genes (DEGs) [95], and the overall differential distribution was displayed via volcano plots. Functional enrichment analyses were performed using Kyoto Encyclopedia of Genes and Genomes (KEGG) pathway analysis and gene set enrichment analysis (GSEA) with the R package ‘clusterProfiler’ (version 4.6.0), with a specific focus on the ferroptosis pathway (mmu04216). The expression profiles of ferroptosis-associated DEGs were visualized in heatmaps based on row-scaled Z scores.

### 5.12 Quantitative real-time polymerase chain reaction (qRT-PCR)

Total RNA was extracted from maxillary bone tissues using TRIzol reagent (Invitrogen, 15596026) according to the established instructions [91]. cDNA was synthesized using Hifair® Ⅲ 1st Strand cDNA Synthesis SuperMix (Yeasen, 11142ES10). Next, qRT-PCR was performed with SYBR Green Master Mix (Yeasen, 11201 ES) on a CFX96 real-time PCR system (Bio-Rad, Hercules, CA, USA). The thermal cycling parameters were set as follows: an initial denaturation step at 95 °C for 30 s, followed by 40 amplification cycles consisting of 95 °C for 10 s and 60 °C for 30 s. A melting curve analysis was carried out to confirm the specificity of the amplified products. The relative mRNA expression levels were normalized to the housekeeping gene *β-actin* and computed using the 2^−ΔΔCt^ method. The primer sequences used for qRT-PCR are listed in Table 1.

**Table 1:** Primer sequences used for qRT-PCR.

Gene	Forward primer sequence (5′–3′)	Reverse primer sequence (5′–3′)
*Gpx4*	GATGGAGCCCATTCCTGAACC	CCCTGTACTTATCCAGGCAGA
*β-actin*	AACAGTCCGCCTAGAAGCAC	CGTTGACATCCGTAAAGACC

Note: *β-actin* was used as the internal control.

### 5.13 TUNEL assay

Cell death was evaluated with a fluorescein-labeled Cell Death Detection Kit (Roche, 11684795910) as previously described [96]. In brief, deparaffinized tissue slices were permeabilized before incubation with the TUNEL reaction system containing terminal deoxynucleotidyl transferase (TdT) and fluorescein-conjugated dUTP. Following rinsing steps, DAPI (Solarbio) was applied to the sections for nuclear counterstaining.

### 5.14 Cell culture

The murine osteocyte-like cell line MLO-Y4 (iCell-m037; iCell Bioscience Inc., Shanghai, China) was cultured in α-MEM (Gibco, 12571063) supplemented with 5% fetal bovine serum (FBS; Brazil origin; Gibco, A5256701), 5% calf serum (CS; Australian origin, Sigma‒Aldrich, B7446), and 1% penicillin–streptomycin solution (Hanheng Bio, HB-PSS-100) at 37 °C in a humidified atmosphere containing 5% CO_2_. Prior to cell seeding, culture plates were precoated with rat tail tendon collagen type I (0.012 mg/mL; Shengyou, 200100) for a minimum of 1 h.

For *in vitro* stimulation, cells were challenged with 100 μM palmitic acid (PA; Kunchuang, KC004) and/or 1 μg/mL *P. gingivalis*-derived LPS (InvivoGen, 14F18-MM) for 24 h. PA was conjugated with bovine serum albumin (BSA; Kunchuang, KC004) as a carrier to increase its solubility. BSA alone (without PA conjugation) served as the carrier control. For pretreatment, taurine (Tau; Sigma-Aldrich, T0625) was added to the culture medium at 5, 10, or 20 mM and incubated with the cells for 6 h prior to the PA/LPS challenge [64,97]. Ferrostatin-1 (Fer-1; 10 μM; Selleck, S7243) and erastin (Era; 10 μM; Selleck, S7242) were added at 10 μM and incubated with the cells for 2 h prior to the treatments [98,99]. Z-Val-Ala-Asp fluoromethylketone (Z-VAD-FMK; MCE,HY-16658B) and necrostatin-1 (Nec-1; MCE, HY-15760) were added at 20 μM and incubated with the cells for 2 h prior to the treatments [100].

### 5.15 Cell Counting Kit-8 (CCK-8) assay

Cell viability was assessed using a Cell Counting Kit-8 (CCK-8; Dojindo, CK04) according to the established instructions [101]. Briefly, MLO-Y4 cells were plated in 96-well plates at a density of 5 × 10^3^ cells per well and incubated overnight for adherence. After the indicated interventions were administered, 10 μL of CCK-8 working solution was added to each well, followed by incubation at 37 °C for 2 h in the dark. The absorbance at 450 nm was determined using a microplate reader (Infinite M200 Pro, Tecan, Switzerland).

### 5.16 C11-BODIPY flow cytometry and malondialdehyde (MDA) measurement

Lipid peroxidation status was assessed by performing C11-BODIPY staining and quantifying MDA levels [102]. Briefly, the cells were incubated with a 2.5 μM C11-BODIPY probe (Thermo Fisher Scientific, D3861) at 37 °C for 30 min under dark conditions. After PBS rinsing, samples were analyzed by flow cytometry (BD Biosciences, San Jose, CA, USA). Oxidized C11-BODIPY fluorescence was detected in the FITC channel, and the data were processed using FlowJo software (v10.8.1; Tree Star, Ashland, OR, USA) for gating and quantification.

In parallel, the level of MDA, a terminal metabolite of lipid peroxidation, was quantified with the Enhanced Cell MDA Colorimetric Assay Kit (Elabscience, E-BC-K814-M) following the product manual. The MDA levels were normalized to the total protein concentration, which was tested using an Omni-Easy™ Ready-to-use BCA Protein Assay Kit (EpiZyme, ZJ102), to correct for differences in cell numbers. The absorbance value at 532 nm was recorded with a microplate reader (Infinite M200 Pro).

### 5.17 Ferrous iron (Fe^2+^) colorimetric assay

Cells were harvested and accurately counted using an automated cell counter (TC20; Bio-Rad Laboratories, Hercules, CA, USA) before lysis to ensure a known cell number. Intracellular Fe^2+^ levels were determined using a Cell Ferrous Iron Colorimetric Assay Kit (Elabscience, E-BC-K881-M) as previously described [103]. The absorbance was recorded at 593 nm via a microplate reader (Infinite M200 Pro). The Fe^2+^ concentrations were calculated based on the standard curve and normalized to cell number, with final results reported as nmol/10^6^ cells.

### 5.18 Western blot analysis

Total protein was extracted from cells using RIPA lysis buffer (Beyotime, P0013B) supplemented with a protease/phosphatase inhibitor cocktail (Beyotime, P1046). Equal amounts of protein were mixed with loading buffer (Affinibody, AIWB-0025). Notably, samples for SLC7A11 detection were incubated sequentially at 37 °C and 70 °C for 5 min each to avoid SLC7A11 aggregation, whereas samples used to detect other proteins were subjected to conventional denaturation at 95 °C for 10 min. The prepared proteins were separated by SDS–PAGE (Epizyme, PG213) and transferred onto 0.22-μm PVDF membranes (Millipore, Billerica, MA, USA) using fast transfer buffer (DINING, DE1400-01). The membranes were blocked with Instant™ Blocking Buffer (Affinibody, AFM-B500) and incubated overnight at 4 °C with primary antibodies against GPX4 (Abmart, T56959; 1:1000), SLC7A11 (Selleck, F0517; 1:1000), ACSL4 (Proteintech, 22401-1-AP; 1:5000), and β-actin (Proteintech, 20536-1-AP; 1:5000). After washes with TBST (ZHHC, PW003) containing Tween-20 (Yeasen, 60305ES76), the membranes were incubated with HRP-conjugated goat anti-rabbit IgG (Proteintech, SA00001-2; 1:5000) for 1 h at room temperature. The protein bands were visualized using ECL reagent (Accuref Sci, AP0080S), and images were captured with a ChemiDoc XRS imaging system (Bio-Rad, Hercules, CA, USA). Band intensities were analyzed via Fiji software (NIH) and normalized to the levels of β-actin.

### 5.19 Transmission electron microscopy (TEM)

Cells (at least 1 × 10^7^) were harvested and centrifuged to form a compact pellet. The pellet was immediately fixed with 3% glutaraldehyde (Sinopharm Chemical Reagent, Shanghai, China) in 0.1 M phosphate buffer at 4 °C for 24 h. After being washed, the samples were postfixed with 1% osmium tetroxide (Zhongjingkeyi Technology, Beijing, China) for 2 h at room temperature, dehydrated through a graded series of acetone solutions (30%, 50%, 70%, 80%, 90%, 95%, and 100%; Xilong Scientific, Shantou, China) and embedded in epoxy resin. Semi-thin sections were stained with toluidine blue (Sinopharm Chemical Reagent) to locate target regions. Ultrathin sections with a thickness of 60–80 nm were prepared and double-stained using uranyl acetate and lead citrate (Zhongjingkeyi Technology). Finally, the ultrastructure of the cells were examined using a JEOL transmission electron microscope (JEM-1400FLASH; JEOL, Tokyo, Japan).

### 5.20 Statistical analysis

All quantitative results are presented as the means ± standard deviations (SDs). Statistical analyses were performed using GraphPad Prism 9.0 (GraphPad Software, San Diego, CA, USA). The unpaired Student's t-test was adopted for comparisons between two groups. One-way analysis of variance (ANOVA) combined with Tukey's or Dunnett's multiple comparison tests was applied for analysis of differences among three or more groups. For histological, IHC, and IF analyses, three representative sagittal sections and three high-power fields per section were evaluated for each animal. All *in vitro* experiments were repeated independently no fewer than three times. For the RNA-seq data, DEGs were identified using DESeq2, with significance defined as unadjusted *P* < 0.05 and |log2 FC| > 0.585. For GSEA, significance was assessed based on the nominal *P* value. A *P* value < 0.05 was regarded as statistically significant.

## Consent for publication

All authors have read and agreed to the published version of the manuscript.

## Funding

The project was funded by the National Natural Science Foundation of China (82301079, 82571070, 82370957, 82401138), the Hong Kong Scholars Program (XJ2025029), the Chang Jiang Scholars Program-Young Scholar (2025), and the Natural Science Foundation of Shaanxi Province (2025JC-YBQN-1042).

## CRediT authorship contribution statement

**Xin-Ge Chen:** Conceptualization, Data curation, Formal analysis, Investigation, Methodology, Validation, Visualization, Writing – original draft. **Xiao-Xue Zhu:** Formal analysis, Investigation, Methodology, Validation. **Cui-Hua Cao:** Data curation, Investigation, Methodology, Validation. **Yu-Zhe Chen:** Data curation, Methodology, Validation. **Yi-Ding Huo:** Investigation, Methodology, Validation. **Dian Gan:** Data curation, Investigation, Validation. **Dao-Kun Deng:** Data curation, Methodology, Validation. **Mei Xu:** Data curation, Funding acquisition. **Hong-Lei Qu:** Funding acquisition, Investigation. **Fa-Ming Chen:** Conceptualization, Funding acquisition, Project administration, Resources, Supervision, Writing – review & editing. **Bei-Min Tian:** Funding acquisition, Project administration, Resources, Supervision, Writing – review & editing. **Xuan Li:** Conceptualization, Funding acquisition, Investigation, Methodology, Project administration, Supervision, Validation, Writing – review & editing.

## Declaration of competing interest

The authors declare no conflicts of interest.

## Data Availability

Data will be made available on request.

## Abbreviations

**3D,** Three-dimensional; **4-HNE,** 4-hydroxynonenal; **AB,** Alveolar bone; **ABC,** Alveolar bone crest; **ACSL4**, Acyl-CoA synthetase long-chain family member 4; **ALT,** Alanine aminotransferase; **ANOVA,** Analysis of variance; **AUC,** Area under the curve; **BHI,** brain heart infusion; **BS,** Bone surface; **BSA,** Bovine serum albumin; **BV/TV,** Bone volume/tissue volume; **C,** Control; **CCK-8,** Cell Counting Kit-8; **CEJ,** Cementoenamel junction; **CFU,** colony-forming units**; CMC,** carboxymethyl cellulose; **CREA,** Creatinine; **CREA-S,** Serum creatinine; **CS,** Calf serum; **DEGs,** Differentially expressed genes; **DIO,** Diet-induced obesity; **DIO-C,** Diet-induced obese control; **DIO-P,** Diet-induced obese with periodontitis; **DMP1,** Dentin matrix protein 1; **Era,** Erastin; **FBS,** Fetal bovine serum; **FDR,** False discovery rate; **Fe^2+^,** Ferrous iron; **Fer-1,** Ferrostatin-1**; FPKM,** Fragments per kilobase of transcript per million mapped reads; ***Fth1***, *Ferritin heavy chain 1 gene*; **FTH1,** Ferritin heavy chain 1; **GPX4,** Glutathione peroxidase 4; **GSEA,** Gene set enrichment analysis; **H&E,** Hematoxylin and eosin; **HFD,** High-fat diet; **IF,** Immunofluorescence; **IHC,** Immunohistochemistry; **IL-4,** Interleukin-4; **IL-6,** Interleukin-6; **KEGG,** Kyoto Encyclopedia of Genes and Genomes; **LC-MS/MS,** Liquid chromatography-tandem mass spectrometry; **LPS,** Lipopolysaccharide; **MDA,** Malondialdehyde; **MFI,** Mean fluorescence intensity; **N.Oc/BS,** Number of osteoclasts per bone surface; **ND,** Normal diet; **Nec-1,** Necrostatin-1; **NES,** Normalized enrichment score **NW,** Normal-weight; **NW-C,** Normal-weight control; **NW-P,** Normal-weight with periodontitis; **OGTT,** Oral glucose tolerance test; **OPG,** Osteoprotegerin; **P,** Periodontitis; ***P. gingivalis*,** *Porphyromonas gingivalis*; **PA,** Palmitic acid; **qRT-PCR,** Quantitative real-time polymerase chain reaction; **RANKL,** Receptor activator of nuclear factor-κB ligand; **RNA-seq,** RNA sequencing; **ROI,** Region of interest; **SD,** Standard deviation; **SOST,** Sclerostin; **Tau,** Taurine; **Tb.N,** Trabecular number; **Tb.Sp,** Trabecular separation; **TdT,** Terminal deoxynucleotidyl transferase; **TEM,** Transmission electron microscopy; **TNF-α,** Tumor necrosis factor-α; **TRAP,** Tartrate-resistant acid phosphatase; **xCT/SLC7A11,** Solute carrier family 7 member 11; **Z-VAD-FMK,** Z-Val-Ala-Asp fluoromethylketone.
