# Supplementary material for: Insights into taurine therapy for periodontitis: Targeting osteocyte ferroptosis to mitigate obesity-exacerbated bone damage
Source: Redox Biol. 2026 Jun 30;95:104282. doi: 10.1016/j.redox.2026.104282 (PMC13352040; doi:10.1016/j.redox.2026.104282)
Supplement: Multimedia component 1 [file mmc1.docx]

## **Appendix A. Supplementary data**

The following are the Supplementary data to this article:


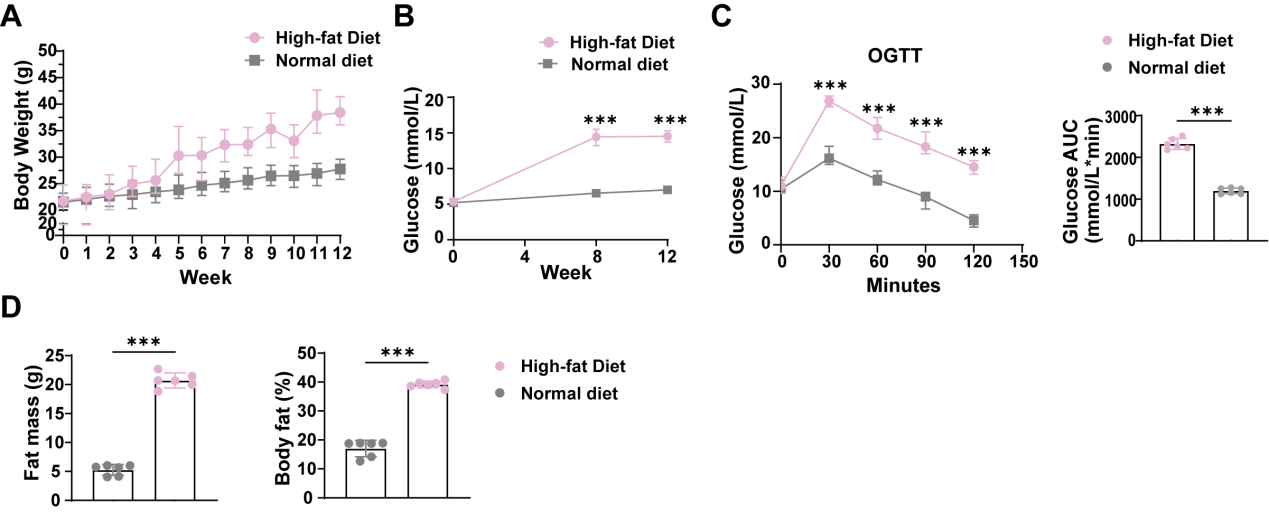


**Supplementary Fig. S1. Establishment and metabolic characterization of the diet-induced obesity (DIO) mouse model. A** Longitudinal body weight changes in mice fed a high-fat diet or a normal diet for 12 weeks (*n* = 40). **B** Blood glucose levels measured at weeks 0, 8, and 12 (*n* = 6). **C** Oral glucose tolerance test (OGTT) performed at week 12; the area under the curve (AUC) was calculated from the OGTT data (*n* = 6). **D** Body composition analysis showing fat mass and body fat percentage at the end of the dietary intervention (*n* = 6). The data are presented as the means ± SDs, and statistical significance was determined using unpaired 2-tailed Student’s t tests. ****P* < 0.001.


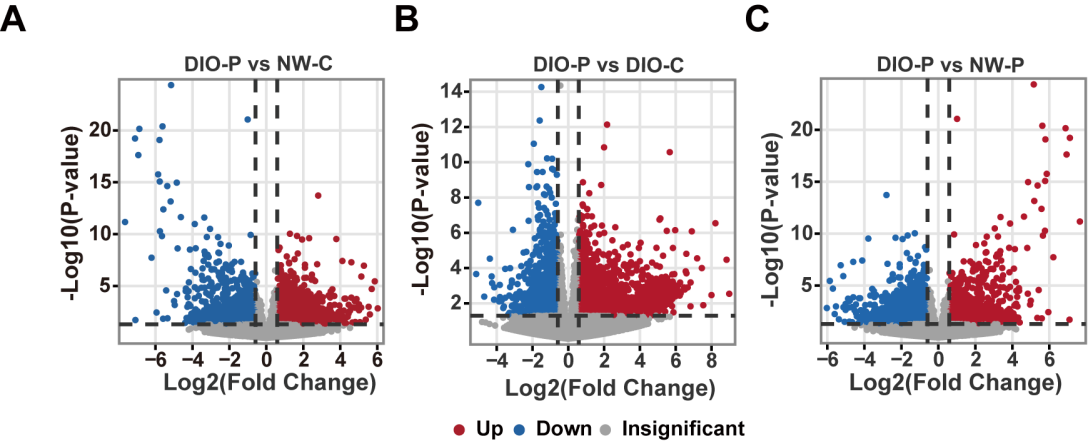


**Supplementary Fig. S2. Identification of differentially expressed genes (DEGs) in the alveolar bone tissues of DIO-P mice compared with those of the other three groups (NW-C, DIO-C, and NW-P mice). A** Volcano plots of DEGs identified in the alveolar bone tissues of DIO-P mice compared with NW-C mice. **B** Volcano plots of DEGs identified in the alveolar bone tissues of DIO-P mice compared with DIO-C mice. **C** Volcano plots of DEGs identified in the alveolar bone tissues of DIO-P mice compared with NW-P mice. Red dots represent genes whose expression is significantly upregulated, blue dots represent genes whose expression is significantly downregulated, and gray dots indicate genes whose expression is not significantly different. n = 5 mice per group.


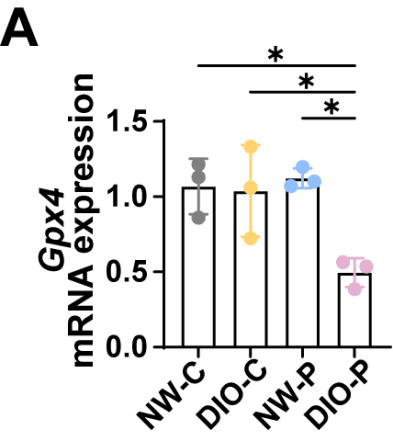


**Supplementary Fig. S3. Validation of *Gpx4* expression in the alveolar bone. A** qPCR validation of *Gpx4* mRNA expression in alveolar bone tissues from NW‑C, DIO‑C, NW‑P, and DIO‑P mice (*n* = 3). The data are presented as the means ± SDs. Statistical significance was determined using one-way ANOVA followed by Dunnett’s multiple comparisons test. **P* < 0.05.

**
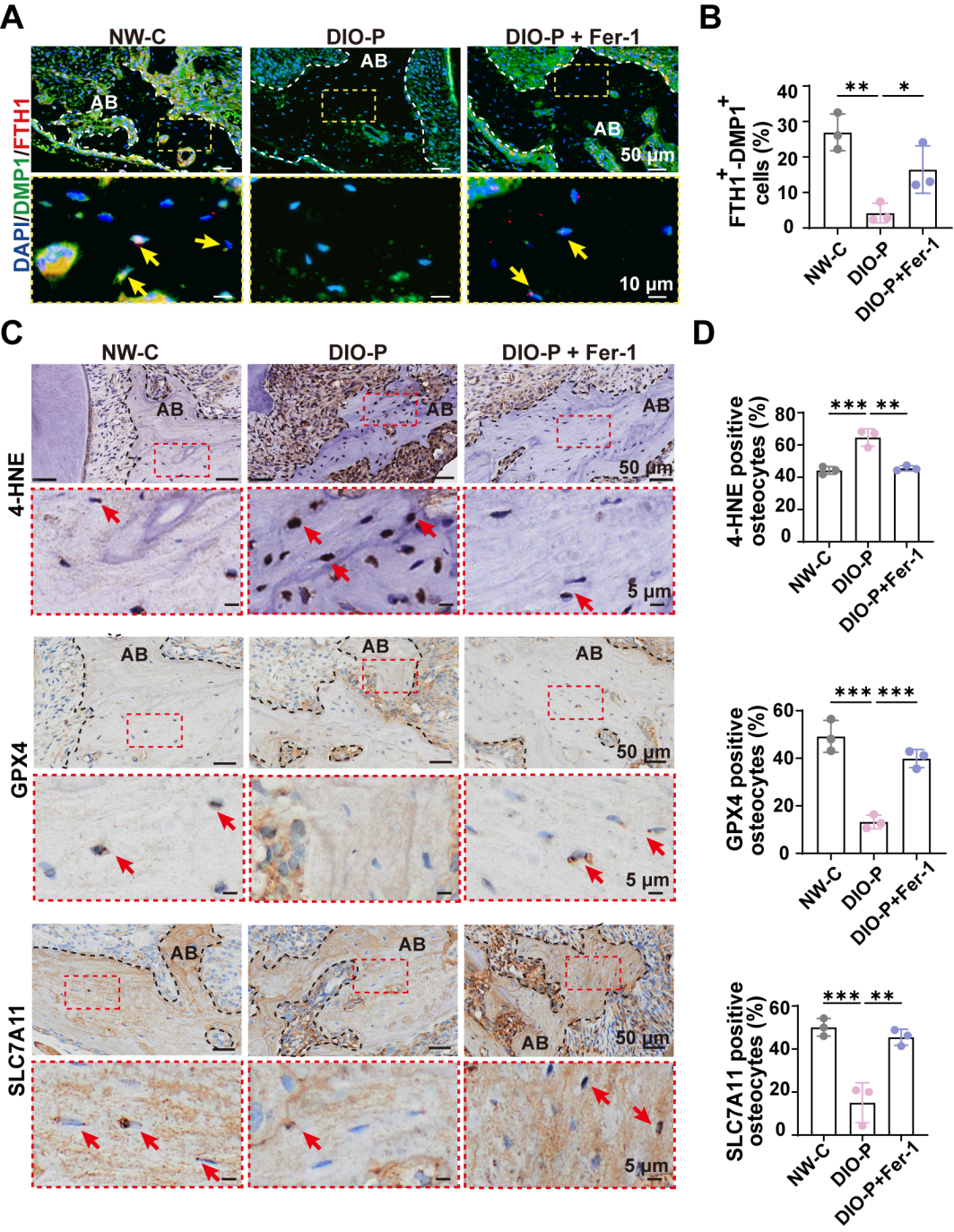
**

**Supplementary Fig. S4. Validation of Fer-1-mediated inhibition of ferroptosis in alveolar bone osteocytes. A** Representative images of IF staining for FTH1 (red) and DMP1 (green) in AB. Yellow dashed boxes indicate enlarged bone regions, and yellow arrows indicate FTH1^+^DMP1^+^cells. Scale bars, 50 μm and 10 μm. **B** Quantification of the percentage of FTH1^+^DMP1^+^cells. **C** Representative images of IHC staining for GPX4, 4-HNE, and SLC7A11 in AB. Red dashed boxes indicate enlarged regions; red arrows indicate positive osteocytes; and black dashed lines delineate the bone boundaries. Scale bars, 50 μm and 5 μm. **D** Quantification of GPX4-, 4-HNE-, and SLC7A11-positive osteocytes in the AB. The data are presented as the means ± SDs. Statistical significance was determined using one-way ANOVA followed by Dunnett’s multiple comparisons test. **P* < 0.05, ***P* < 0.01, and ****P* < 0.001. *n* = 3 mice per group.

**
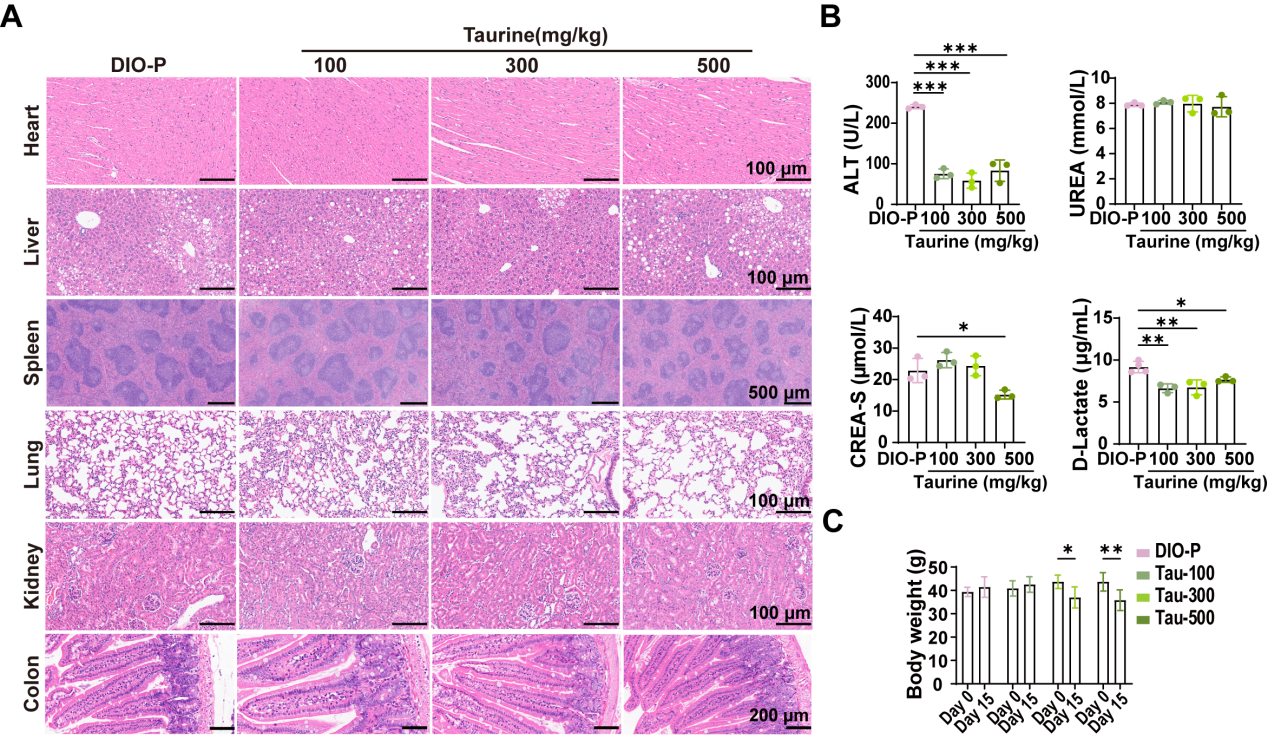
Supplementary Fig. S5. Systemic safety assessment and body weight monitoring in taurine-treated DIO-P mice. A** Representative images of H&E-stained sections of major organs, including the heart, liver, spleen, lung, kidney, and colon, from DIO-P mice receiving taurine supplementation via combined oral gavage and drinking water administration. Taurine was administered by oral gavage twice daily at 100, 300, or 500 mg/kg and simultaneously provided in the drinking water at 0.1%, 0.2%, or 0.4%, respectively. Scale bars, 100 μm (heart, liver, lung, and kidney), 500 μm (spleen), and 20 μm (colon). **B** Serum levels of biochemical parameters for an evaluation of systemic safety and the gut barrier status, including alanine aminotransferase (ALT; liver function), urea, creatinine (CREA; kidney function), and D-lactate (a marker of increased intestinal permeability), in DIO-P mice after taurine supplementation at the indicated doses. **C** Body weight of DIO-P mice receiving taurine supplementation at the indicated doses via combined oral gavage and drinking water administration. The data are presented as the means ± SDs. Statistical significance was determined using one-way ANOVA followed by Dunnett’s multiple comparisons test. **P* < 0.05, ***P* < 0.01, and ****P* < 0.001. *n* = 3 mice per group.


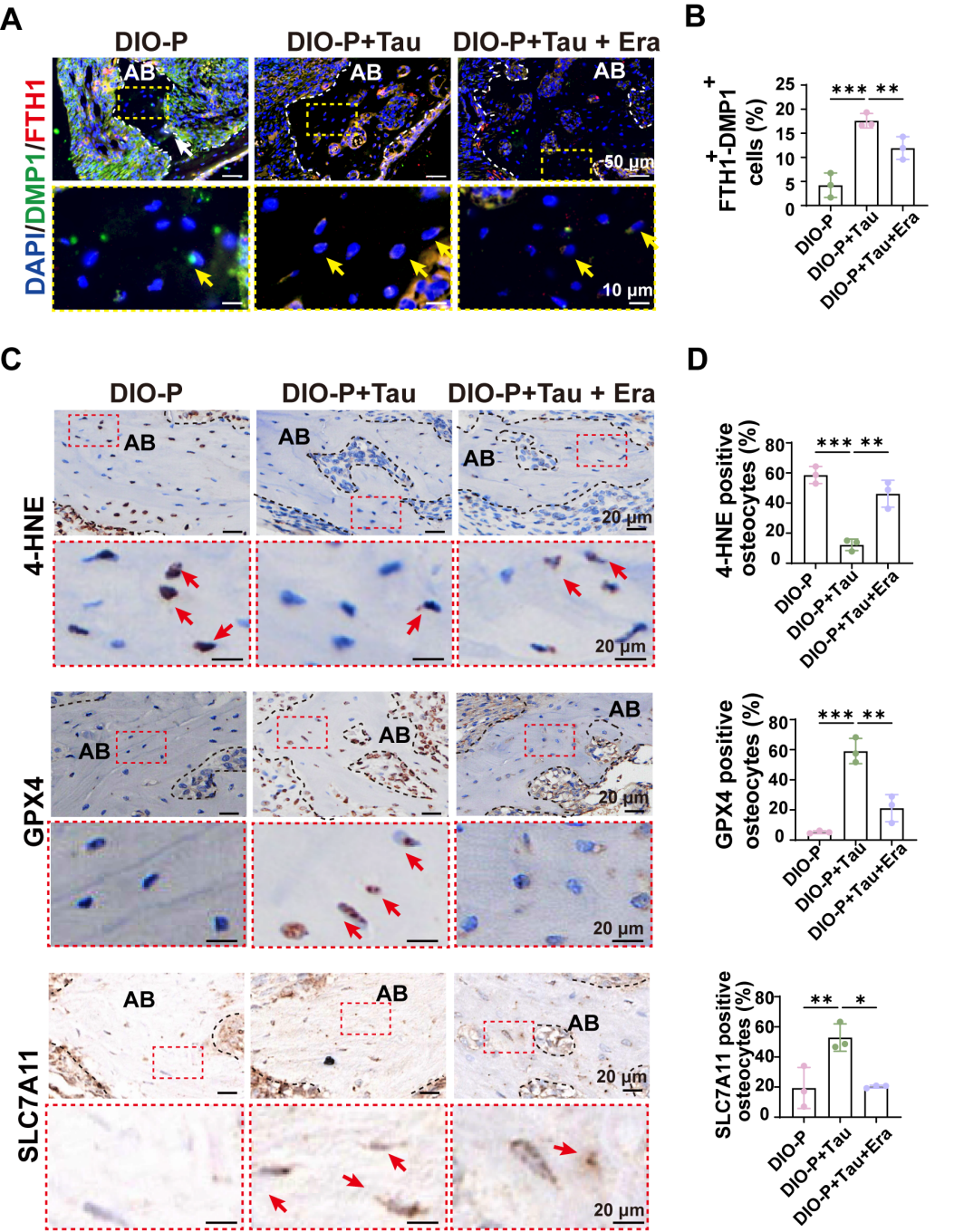


**Supplementary Fig. S6. Validation of erastin-mediated activation of ferroptosis in alveolar bone osteocytes. A** Representative images of IF staining for FTH1 (red) and DMP1 (green) in AB. Yellow dashed boxes indicate enlarged bone regions, and yellow arrows indicate FTH1^+^DMP1^+^cells. Scale bars, 50 μm and 10 μm. **B** Quantification of the percentage of FTH1^+^ DMP1^+^cells. **C** Representative images of IHC staining for GPX4, 4-HNE, and SLC7A11 in AB. Red dashed boxes indicate enlarged regions; red arrows indicate positive osteocytes; and black dashed lines delineate the bone boundary. Scale bars, 20 μm. **D** Quantification of GPX4-, 4-HNE-, and SLC7A11-positive osteocytes in AB. The data are presented as the means ± SDs. Statistical significance was determined using one-way ANOVA followed by Dunnett’s multiple comparisons test. **P* < 0.05, ***P* < 0.01, and ****P* < 0.001. *n* = 3 mice per group.
